# Supplementary material for: Unique Interaction between Layered Black Phosphorus and Nitrogen Dioxide
Source: Nanomaterials (Basel). 2022 Jun 10;12(12):2011. doi: 10.3390/nano12122011 (PMC9230001; doi:10.3390/nano12122011)
Supplement: Supplementary file 1 [file nanomaterials-12-02011-s001.zip › nanomaterials-1709247-supplementary.pdf]

nanomaterials

Supporting Information

Unique Interaction between Layered Black Phosphorus and Nitrogen Dioxide

Jingjing Zhao [1,2], Xuejiao Zhang [1], Qing Zhao [1,3,4], Xue-Feng Yu [5], Siyu Zhang [1,*] and Baoshan Xing [6]

1 Key Laboratory of Pollution Ecology and Environmental Engineering, Institute of Applied Ecology, Chinese Academy of Sciences, Shenyang 110016, China; zjj911051836@126.com (J.Z.); zhangxuejiao@iae.ac.cn (X.Z.); zhaoqing@iae.ac.cn (Q.Z.)
2 Shenyang Institute of Applied Ecology, University of Chinese Academy of Sciences, Beijing 100049, China
3 Key Laboratory of Integrated Agro-Environmental Pollution Control and Management, Institute of Eco-Environmental and Soil Sciences, Guangdong Academy of Sciences, Guangzhou 510650, China
4 China National-Regional Joint Engineering Research Center for Soil Pollution Control and Remediation in South China, Guangzhou 510650, China
5 Materials and Interfaces Center, Shenzhen Institutes of Advanced Technology, Chinese Academy of Sciences, Shenzhen 518055, China; xf.yu@siat.ac.cn
6 Stockbridge School of Agriculture, University of Massachusetts, Amherst, MA 01003, USA; bx@umass.edu
* Correspondence: syzhang@iae.ac.cn; Tel.: +86-24-8397-0383

Citation: Zhao, J.; Zhang, X.; Zhao, Q.; Yu, X.-F.; Zhang, S.; Xing, B. Unique Interaction between Layered Black Phosphorus and Nitrogen Dioxide. *Nanomaterials* **2022**, *12*, 2011. https://doi.org/10.3390/nano12122011

Academic Editor: Yu-Chun Chiang

Received: 18 April 2022
Accepted: 6 June 2022
Published: 10 June 2022

Publisher's Note: MDPI stays neutral with regard to jurisdictional claims in published maps and institutional affiliations.

Copyright: © 2022 by the authors. Licensee MDPI, Basel, Switzerland. This article is an open access article distributed under the terms and conditions of the Creative Commons Attribution (CC BY) license (https://creativecommons.org/licenses/by/4.0/).

Table of Contents

Figure S1. Valence electrons in the outmost valence shell of gas molecules: (a) NO_2, (b) SO_2, (c) CO_2, (d) NO, (e) CO and (f) NH_3. 1
Figure S2. Honeycomb wrinkled atomic structural diagram of SLBP. 1
Figure S3. Variations of E_{ad} (eV) with $d_{shortest}$ (Å) or orientations of gas molecule: (a) NO_2, (b) SO_2, (c) CO_2, (d) NO, (e) CO and (f) NH_3. 2
Figure S4. Variations of E_{ad} (eV) with $d_{shortest}$ (Å) or types of adsorption sites in fully-optimized configurations of adsorption complexes: (a) NO_2, (b) SO_2, (c) CO_2, (d) NO, (e) CO and (f) NH_3. 2
Figure S5. Side and top views of thermodynamically favorable adsorption configurations of gas molecules on SLBP: (a) NO_2−**2O*vz***, (b) NO_2−**N*va***, (c) NO_2−**N*vz***, (d) SO_2−***pz***, (e) SO_2−**S*vz***, (f) SO_2−***pa***, (g) CO_2−***pa***, (h) NO−***pa***, (i) CO−***pa***, (j) CO−***pa*** and (k) NH_3−**N*va***. 3
Figure S6. Modified interaction energies (E_{ad-def}, eV) of the lowest-energy adsorption configurations calculated for NO_2, SO_2 or CO_2 on d−SLBP. 4
Figure S7. Side and top views of the energetically stable adsorption configurations calculated for NO (a ~ d) and NH_3 (e ~ h) on SV (a, e), DV3 (b, f), EGz1 (c, g) and EGz2 (d, h). 4
Figure S8. TDOS of adsorption complexes of NO and d−SLBP and PDOS of d−SLBP in adsorption complexes. (a) SV, (b) DV3, (c) EGz1 and (d) EGa2. 5
Figure S9. Initial configurations of SLBP and d−SLBP: (a) SLBP, (b) SW1, (c) SW2, (d) SV, (e) DV1, (f) DV2, (g) DV3, (h) EGz1, (i) EGz2, (j) EGa1 and (k) EGa2. 6
Figure S10. Initial configurations of NO_2 molecule adsorbed onto SLBP: (a) **1O*v***, (b) **N*va***, (c) **N*vz***, (d) **2O*va***, (e) **2O*vz***, (f) ***pa***, (g) ***pz***. 6
Figure S11. Initial configurations of SO_2 molecule adsorbed onto SLBP: (a) **1O*v***, (b) **S*va***, (c) **S*vz***, (d) **2O*va***, (e) **2O*vz***, (f) ***pa***, (g) ***pz***. 7
Figure S12. Initial configurations of CO_2 molecule adsorbed onto SLBP: (a) **O*v***, (b) ***pa***, (c) ***pz***. 7
Figure S13. Initial configurations of NO molecule adsorbed onto SLBP: (a) **N*v***, (b) **O*v***, (c) ***pa***, (d) ***pz***. 7
Figure S14. Initial configurations of CO molecule adsorbed onto SLBP: (a) **C*v***, (b) **O*v***, (c) ***pa***, (d) ***pz***. 8
Figure S15. Initial configurations of NH_3 molecule adsorbed onto SLBP: (a) **1H*v***, (b) **3H*v***, (c) **N*va***, (d) **N*vz***. 8
Figure S16. Convergence tests of cutoff energy (a) and k−point grid (b). 9
Figure S17. Side and top views of the most energetically stable adsorption configurations calculated for NO_2 and d−SLBP. (a) SW1−NO_2, (b) SW2−NO_2, (c) SV−NO_2, (d) DV1−NO_2, (e) DV2−NO_2, (f) DV3−NO_2, (g) EGz1−NO_2, (h) EGz2−NO_2, (i) EGa1−NO_2 and (j) EGa2−NO_2. 15
Figure S18. Side and top views of the most energetically stable adsorption configurations of SO_2 on d−SLBP. (a) SW1−SO_2, (b) SW2−SO_2, (c) SV−SO_2, (d) DV1−SO_2, (e) DV2−SO_2, (f) DV3−SO_2, (g) EGz1−SO_2, (h) EGz2−SO_2, (i) EGa1−SO_2 and (j) EGa2−SO_2. 16
Figure S19. Side and top views of the most energetically stable adsorption configurations of CO_2 on d−SLBP. (a) SW1−CO_2, (b) SW2−CO_2, (c) SV−CO_2, (d) DV1−CO_2, (e) DV2−CO_2, (f) DV3−CO_2, (g) EGz1−CO_2, (h) EGz2−CO_2, (i) EGa1−CO_2 and (j) EGa2−CO_2. 17
Figure S20. Side and top views of the desorption configuration. 19
Figure S21. Side and top views of deformed DV3 after adsorption. 20
Figure S22. Total density of states (TDOS) of SLBP and d−SLBP and partial DOS (PDOS) of key phosphorus atoms contributing to changes of total DOS. (a) Atom $\mathbf{P}_{17}$ in SLBP, (b) atom $\mathbf{P}_{17}$ in SW1, (c) atom $\mathbf{P}_{17}$ in SW2, (d) atom $\mathbf{P}_{17}$ in SV, (e) atom $\mathbf{P}_{17}$ in DV1, (f) atom $\mathbf{P}_{20}$ in DV2, (g) atom $\mathbf{P}_{17}$ in DV3, (h) atom $\mathbf{P}_{31}$ in EGz1, (i) atom $\mathbf{P}_{27}$ in EGz2, (j) atom $\mathbf{P}_{21}$ in EGa1 and (k) atom $\mathbf{P}_{11}$ in EGa2. 22
Figure S23. Band structures and DOS patterns of SLBP and gas adsorption complexes. (a) NO_2, (b) SO_2, (c) CO_2, (d) NO, (e) CO, (f) NH_3. 23
Figure S24. TDOS of adsorption complexes of d−SLBP and NO_2 and PDOS of d−SLBP in adsorption complexes. (a) SW1, (b) SW2, (c) SV, (d) DV1, (e) DV2, (f) DV3, (g) EGz1, (h) EGz2, (i) EGa1 and (j) EGa2. 24
Figure S25. TDOS of adsorption complexes of d−SLBP and SO_2 and PDOS of d−SLBP in adsorption complexes. (a) SW1, (b) SW2, (c) SV, (d) DV1, (e) DV2, (f) DV3, (g) EGz1, (h) EGz2, (i) EGa1 and (j) EGa2. 25
Figure S26. TDOS of adsorption complexes of CO_2 and d−SLBP and PDOS of d−SLBP in adsorption complexes. (a) SW1, (b) SW2, (c) SV, (d) DV1, (e) DV2, (f) DV3, (g) EGz1, (h) EGz2, (i) EGa1 and (j) EGa2. 26

Figure S27. Characterizations of LBP. (a) Raman spectrum of LBP; (b) TEM image of LBP. 28
Table S1. Formation energies (E_f, eV) of defects in LBP and graphene 13
Table S2. Adsorption energies (E_{ad}, eV), E_{vdW}/E_{ad} (%), the shortest distances ($d_{shortest}$, Å) and charge transfer (Δq, e) of the lowest–energy adsorption configurations of NO_2, SO_2 and CO_2 on SLBP and each d–SLBP 18
Table S3. Charge and spin population on each atom in the desorption configuration of SV absorbing NO_2 by Mulliken Atomic Populations 19
Table S4. Interaction energies (E_{ad}, eV), deformation energies (E_{def}, eV) and modified interaction energies (E_{ad-def}, eV) for d–SLBP and dioxides 21

Text Table of Contents

S1. Initially Constructed Adsorbate and Adsorption Complex Configurations 5
S2. Calculation Method 8
S3. Computational Results on Adsorption of Gas Molecules on Perfect SLBP 10
S4. The Most Stable Configuration of Gas Molecule on SLBP and Related Disputes 10
S5. Computational Results on Individual d–SLBP 14
S6. Computational Results on Adsorption of Gas Molecules on d–SLBP 14
S7. Nature of the **[NO]** Species 19
S8. Deformation of DV3 after interacting with gas molecules 20
S9. Single Electron Dominated Mechanism for SLBP or d-SLBP Absorbing Gas Molecules 21
S10. Experimental Details 27

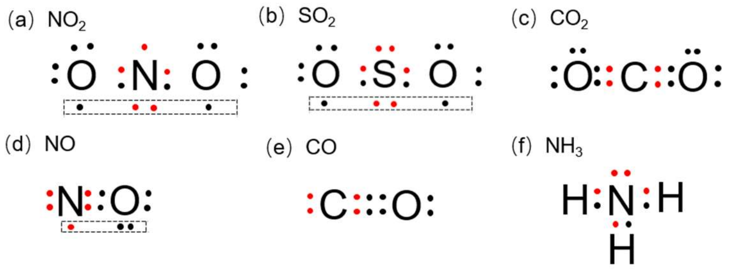

Figure S1. Valence electrons in the outmost valence shell of gas molecules: (a) NO_2, (b) SO_2, (c) CO_2, (d) NO, (e) CO and (f) NH_3. Red and black dots represent valence electrons. A dashed box refers to a special bonding type.

Figure S1 shows outmost valence electrons of gas molecules. The valence bond theory has no clear conclusion on the electronic structure of NO_2. The most accepted point of view is that NO_2 has a three-center, four-electron π bond [1]. Nitrogen is sp^2 hybridized in NO_2. Two of three sp^2 orbital electrons of NO_2 form two N–O single bonds with *p* orbital electrons of the oxygen atom, and the last sp^2 electron is unpaired. The other two paired valence electrons of the nitrogen atom and two electrons from two oxygen atoms form a four-electron, three-center π bond (Figure S1a). SO_2 has a four-electron, three-center π bond containing no single electron (Figure S1b). Two ***sp*** hybrid orbitals of carbon atoms form two σ bonds with two oxygen atoms in CO_2 (Figure S1c). NO has an odd number of valence electrons. Differently from NO_2, the single electron of NO is in a two-center three-electron bond (Figure S1d) [2]. In CO, two electrons of the carbon atom and two electrons of the oxygen atom form two covalent bonds, and the lone pair electron of the oxygen atom and an empty *p* orbital of the carbon atom form a coordinate bond, forming a total of three covalent bonds (Figure S1e) [3]. In NH_3, the nitrogen atom has five valence electrons, of which the three unpaired combine with three hydrogen atoms (Figure S1f).

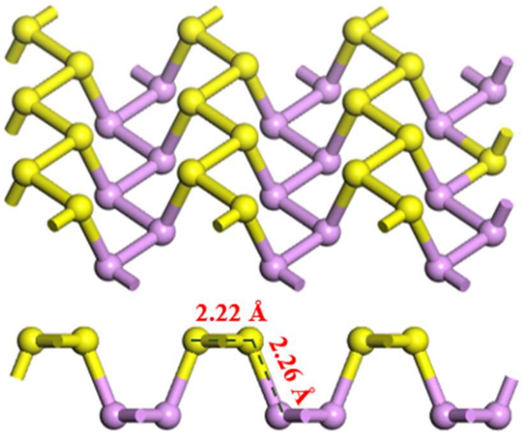

Figure S2. Honeycomb wrinkled atomic structural diagram of SLBP.

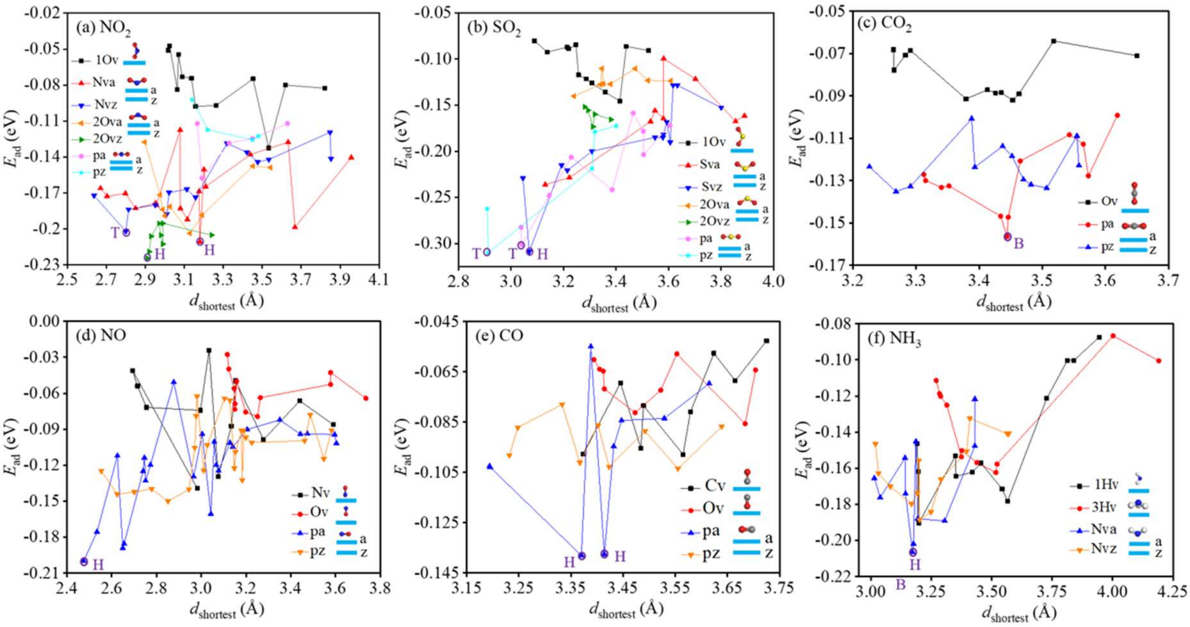

Figure S3. Variations of E_{ad} (eV) with $d_{shortest}$ (Å) or orientations of gas molecule: (a) NO_2, (b) SO_2, (c) CO_2, (d) NO, (e) CO and (f) NH_3. **1O*v***: one of two oxygen atoms in nonlinear NO_2 and SO_2 points to SLBP. **N*va*** (**S*va***) or **N*vz*** (**S*vz***): nitrogen (sulfur) atom of nonlinear NO_2 or NH_3 (SO_2) points to the SLBP and the molecule is an armchair or zigzag shape. **2O*va*** or **2O*vz***: two oxygen atoms of nonlinear NO_2 and SO_2 point to SLBP and the molecule is an armchair or zigzag shape. ***pa*** or ***pz***: a gas molecule is parallel to the SLBP plane in armchair or zigzag shape. **O*v***, **N*v*** or **C*v***: single oxygen, nitrogen or carbon atom of linear CO_2, NO or CO points to the SLBP plane. **1H*v*** or **3H*v***: 1 or 3 hydrogen atoms of NH_3 point to the SLBP plane.

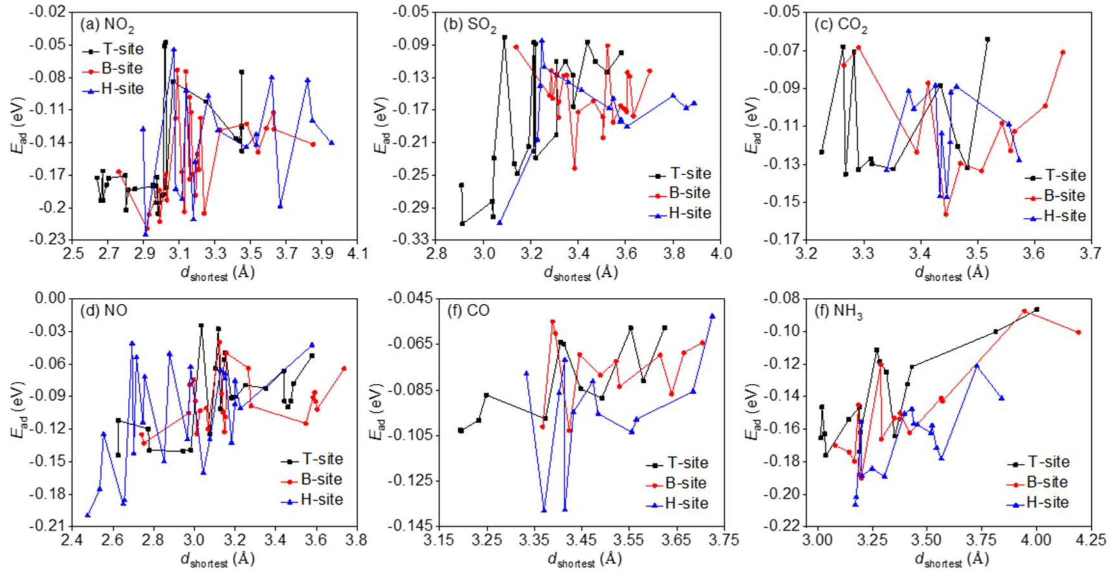

Figure S4. Variations of E_{ad} (eV) with $d_{shortest}$ or types of adsorption sites in fully-optimized configurations of adsorption complexes: (a) NO_2, (b) SO_2, (c) CO_2, (d) NO, (e) CO and (f) NH_3.

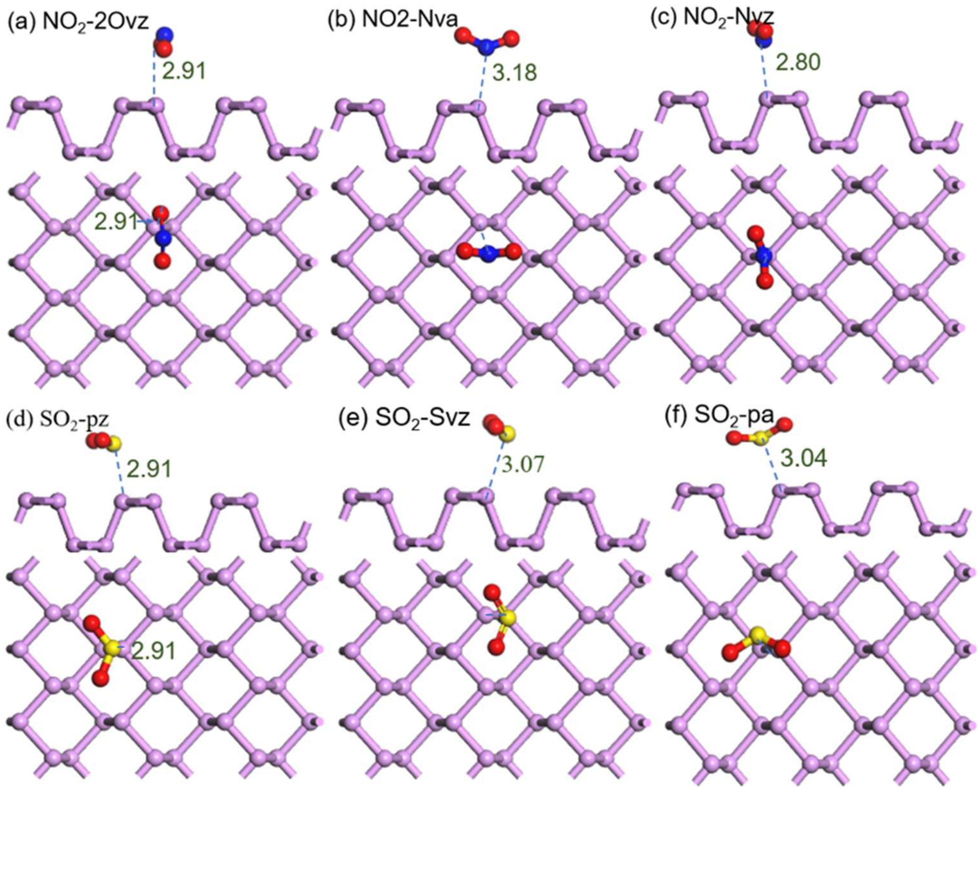

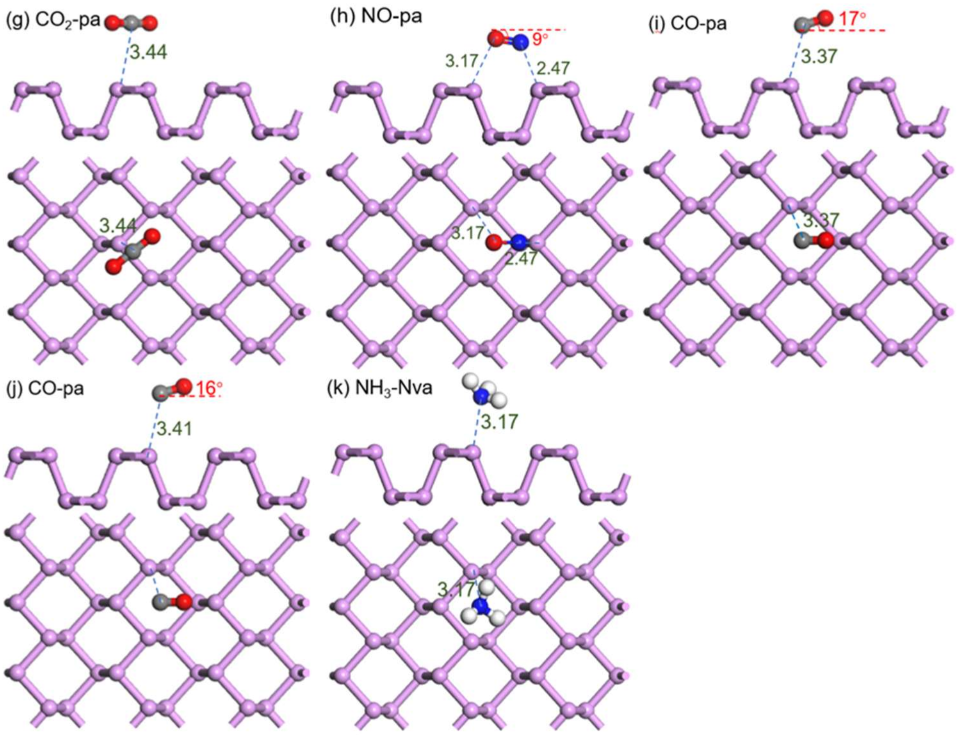

Figure S5. Side and top views of thermodynamically favorable adsorption configurations of gas molecules on SLBP: (a) NO_2−**2O*vz***, (b) NO_2−**N*va***, (c) NO_2−**N*vz***, (d) SO_2−***pz***, (e) SO_2−**S*vz***, (f) SO_2−***pa***, (g) CO_2−***pa***, (h) NO−***pa***, (i) CO−***pa***, (j) CO−***pa*** and (k) NH_3−**N*va***. Green numbers represent $d_{shortest}$ values between SLBP and gases. Red numbers are dihedral angles between the gas and the SLBP plane.

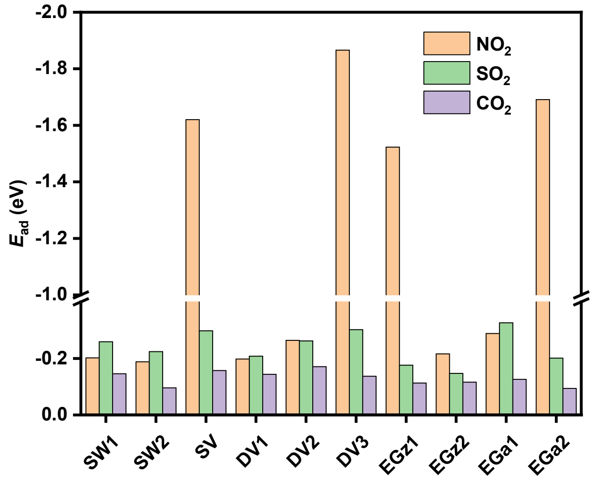

Figure S6. Modified interaction energies (E_{ad-def}, eV) of the lowest-energy adsorption configurations calculated for NO_2, SO_2 or CO_2 on d−SLBP.

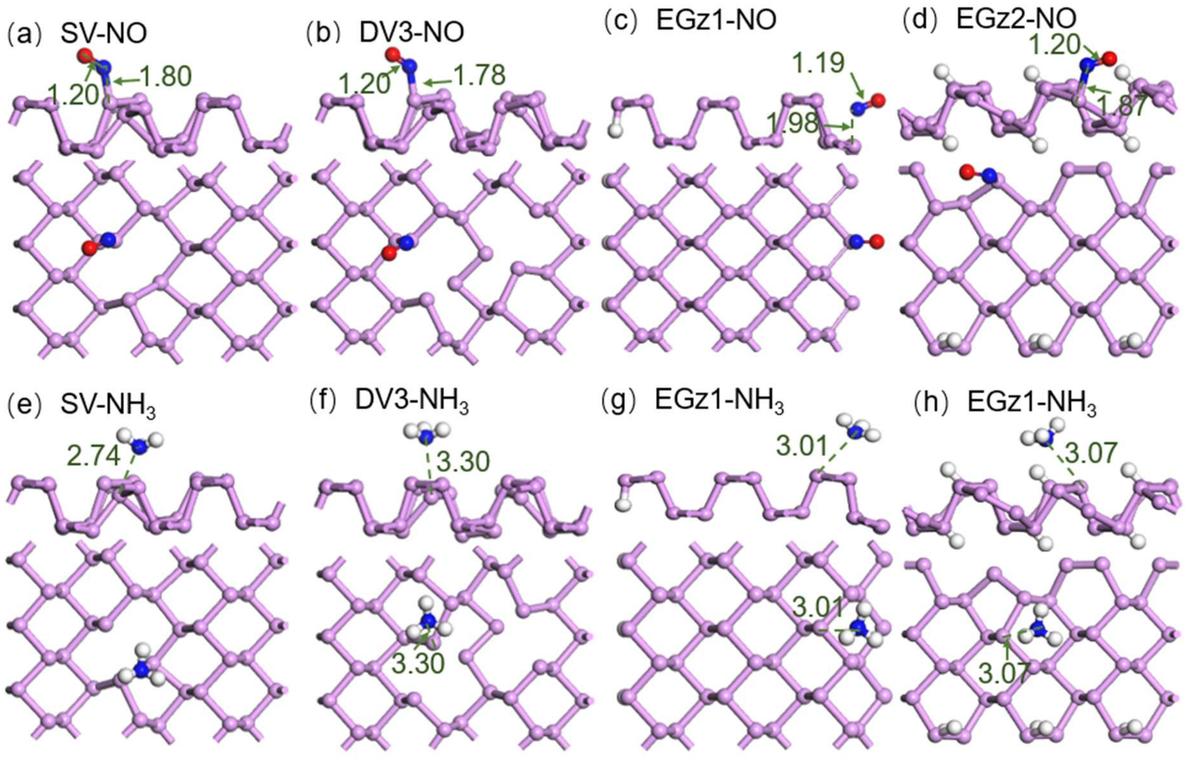

Figure S7. Side and top views of the energetically stable adsorption configurations calculated for NO (a ~ d) and NH_3 (e ~ h) on SV (a, e), DV3 (b, f), EGz1 (c, g) and EGz2 (d, h). Green numbers: the shortest atomic distances ($d_{shortest}$) between d-SLBP and gas molecules.

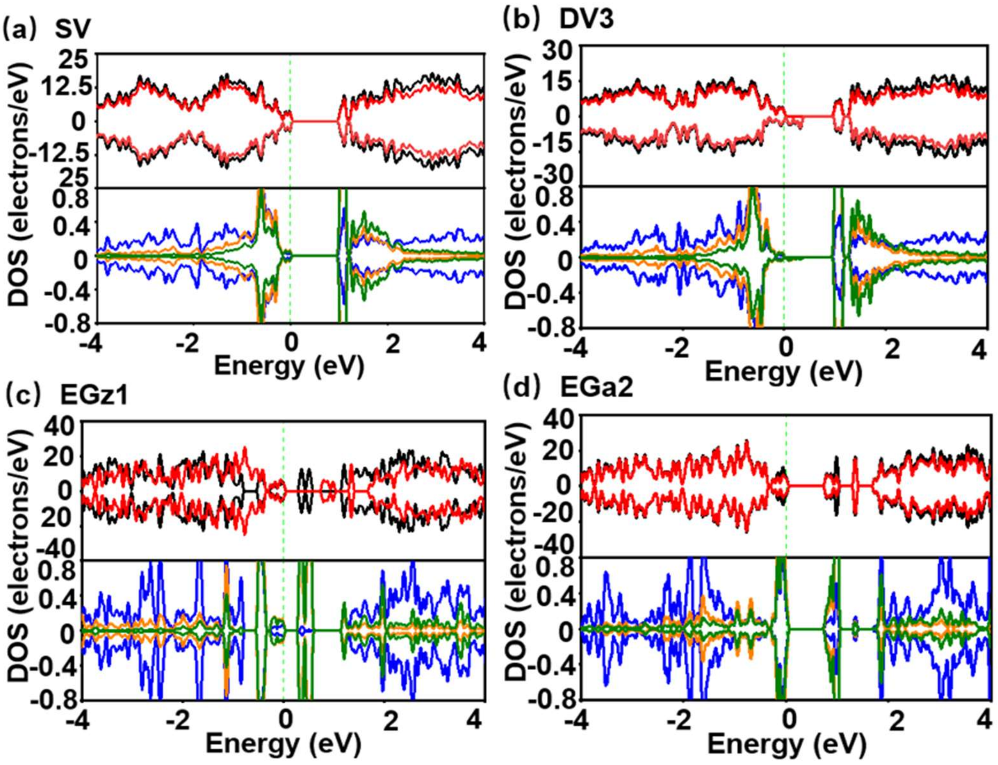

Figure S8. TDOS of adsorption complexes of NO and d−SLBP (black solid line) and PDOS of d−SLBP (red solid line) in adsorption complexes (upper panel). (a) SV, (b) DV3, (c) EGz1 and (d) EGa2. PDOS of *p* orbitals of oxygen (orange solid line) and nitrogen (dark green solid line) atoms and the phosphorus atom (blue solid line) nearest to NO (lower panel). Green dashed line: Fermi level.

S1. Initially Constructed Adsorbate and Adsorption Complex Configurations

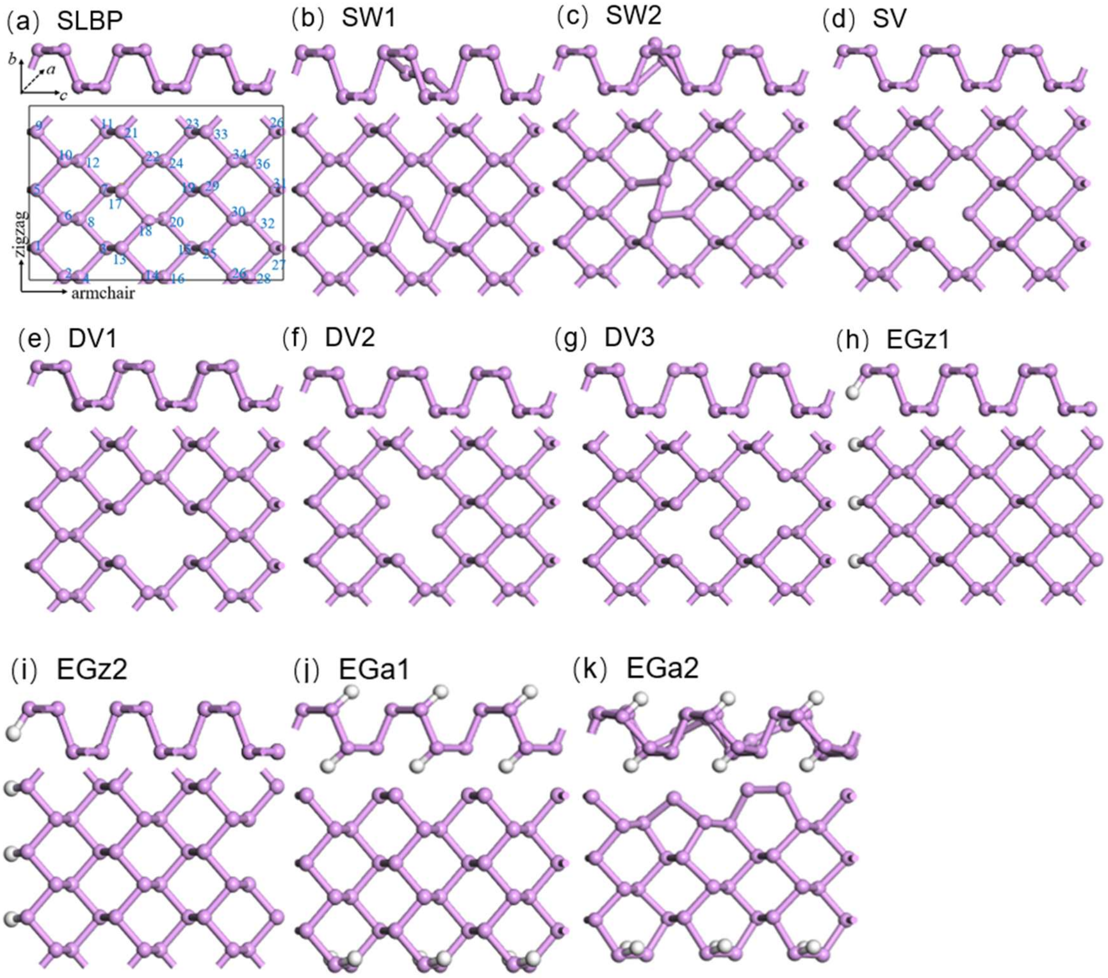

Figure S9. Initial configurations of SLBP and d-SLBP: (a) SLBP, (b) SW1, (c) SW2, (d) SV, (e) DV1, (f) DV2, (g) DV3, (h) EGz1, (i) EGz2, (j) EGa1 and (k) EGa2.

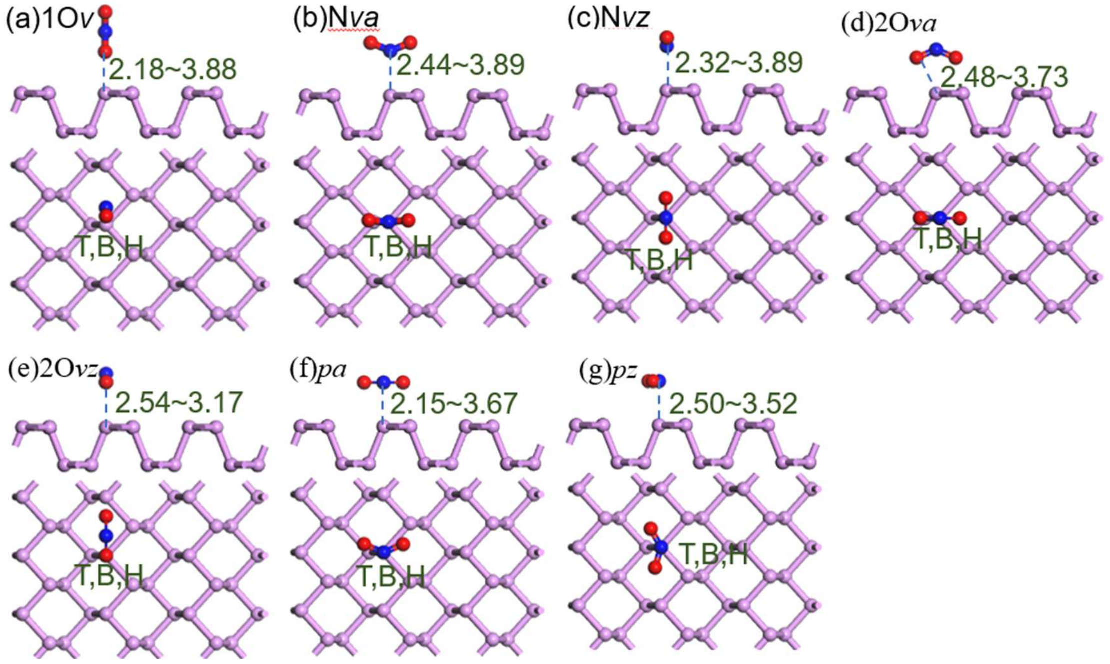

Figure S10. Initial configurations of NO_2 molecules adsorbed onto SLBP: (a) **1O*v***, (b) **N*va***, (c) **N*vz***, (d) **2O*va*,** (e) **2O*vz*,** (f) ***pa*,** (g) ***pz***.

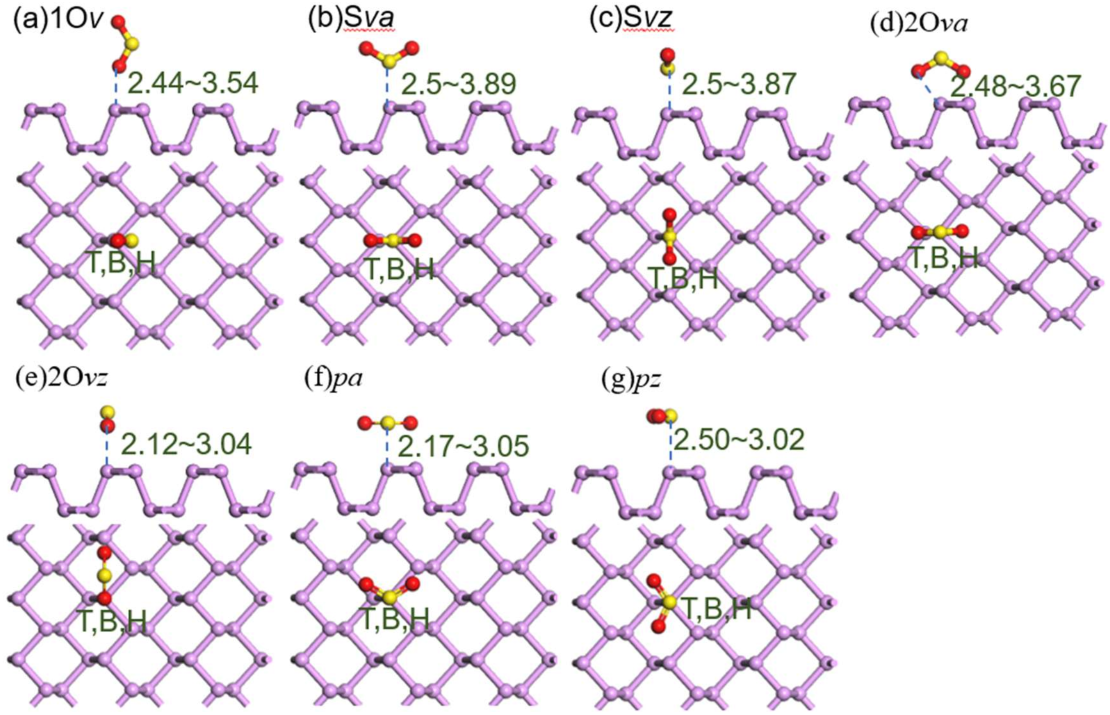

Figure S11. Initial configurations of SO_2 molecules adsorbed onto SLBP: (a) **1O*v***, (b) **S*va***, (c) **S*vz***, (d) **2O*va*,** (e) **2O*vz*,** (f) ***pa*,** (g) ***pz***.

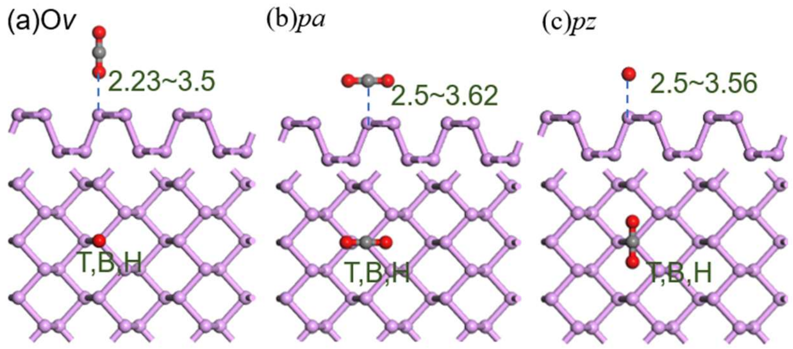

Figure S12. Initial configurations of CO_2 molecules adsorbed onto SLBP: (a) **O*v***, (b) ***pa,*** (c) ***pz***.

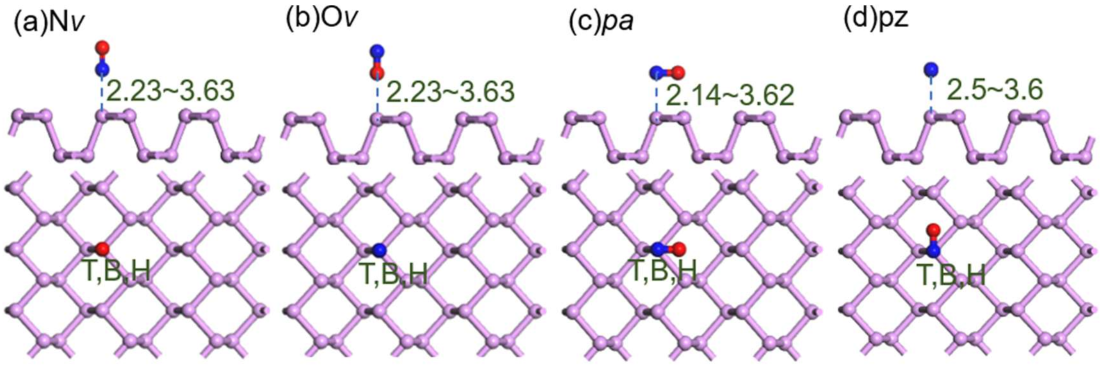

Figure S13. Initial configurations of NO molecules adsorbed onto SLBP: (a) **N*v*,** (b) **O*v***, (c) ***pa*** (d) ***pz***.

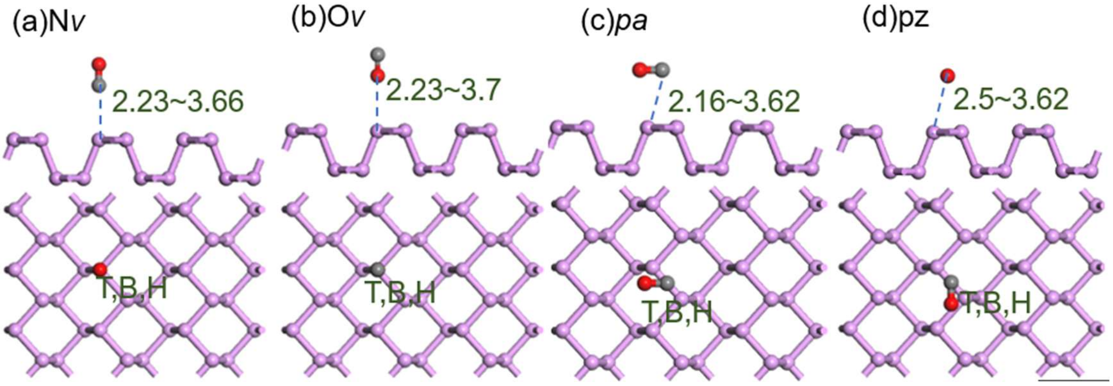

Figure S14. Initial configurations of CO molecules adsorbed onto SLBP: (a) **C*v*** (b) **O*v***, (c) ***pa,*** (d) ***pz***.

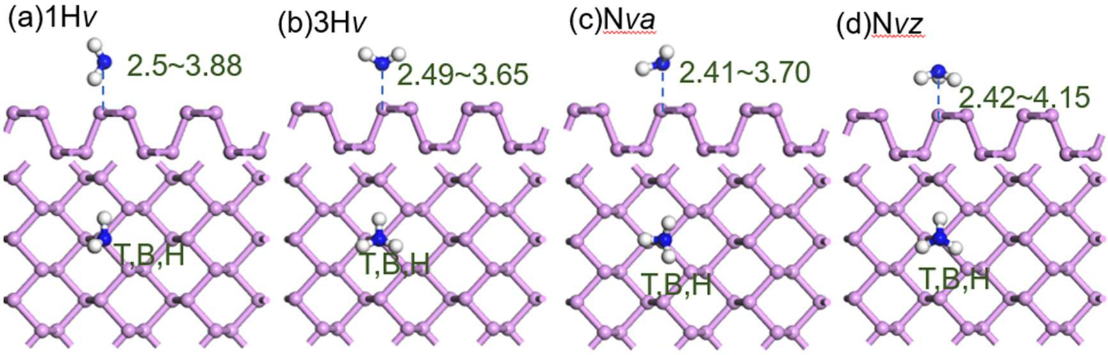

Figure S15. Initial configurations of NH_3 molecules adsorbed onto SLBP: (a) **1H*v***, (b) **3H*v***, (c) **N*va***, (d) **N*vz***.

S2. Calculation Method

In order to ensure the accuracy of the calculation parameters, convergence tests of cutoff energy and k-point were carried out. At k-point 4 × 1 × 3, the cutoff energy was set to 250-700 eV for testing. Figure S16a shows that energy convergence is reached when the cutoff energy is 500 eV. Then, the cutoff energy was set to 500 eV, and different k-points (4 × 1 × 3, 8 × 1 × 6, 10 × 1 × 8) were tested. Figure S16b shows that a k-point larger than 4 × 1 × 3 cannot induce an impact on energy. Therefore, the cutoff energy and k-point in geometry optimizations or property computations except for DOS were set to 500 eV and 4 × 1 × 3, respectively.

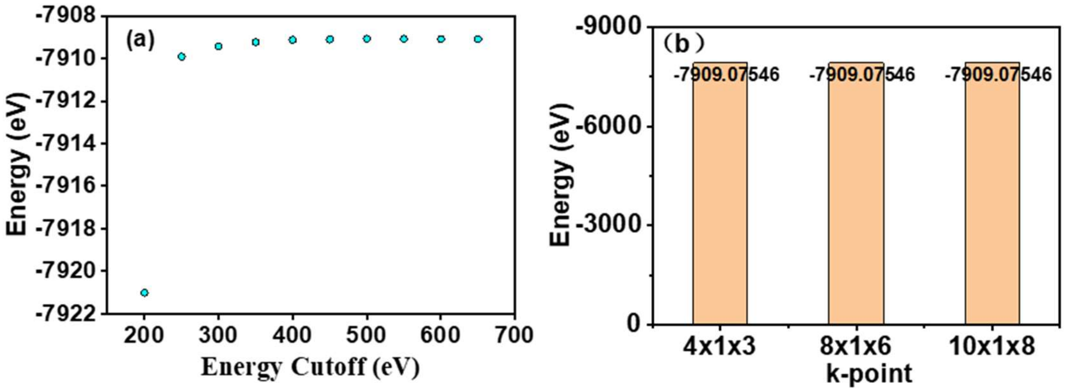

Figure S16. Convergence tests of cutoff energy (a) and k−point grid (b).

The $d_{shortest}$ between adsorbed gas molecules and phosphorus atoms in fully-optimized adsorption configuration was measured to evaluate interaction strength. Adsorption energies including (E_{ad}) and not including (E_0) vdW interaction energy (E_{vdW}) corrections are calculated following Equations (1) and (2), respectively:

$$E_{ad} = E_{complex} - E_{SLBP/d\text{-}SLBP} - E_{gas} \quad (1)$$

$$E_0 = E'_{complex} - E'_{SLBP/d\text{-}SLBP} - E'_{gas} \quad (2)$$

where $E_{complex}$, $E_{SLBP/d\text{-}SLBP}$ and E_{gas} are total energies including vdW corrections of adsorption complex, SLBP (or d-SLBP) and a gas molecule, respectively; $E'_{complex}$, $E'_{SLBP/d\text{-}SLBP}$ and E'_{gas} represent total energies without vdW correction. A negative E_{ad} value means that the adsorption process is exothermic. An adsorption complex possessing a large negative E_{ad} value is thermodynamically more stable than one having a small negative E_{ad} value. E_{vdW} is calculated following Equation (3).

$$E_{vdW} = E_{ad} - E_0 \quad (3)$$

E_{def} of d-SLBP after adsorbing gas molecules are obtained following Equation (4). Then, E_{ad} is corrected by the E_{def} values following Equation (5) to generate modified interaction energy ($E_{ad\text{-}def}$) for a d-SLBP adsorbing gas molecule.

$$E_{def} = E_{d\text{-}SLBP(def)} - E_{d\text{-}SLBP} \tag{4}$$

$$E_{ad\text{-}def} = E_{ad} - E_{def} \tag{5}$$

where $E_{d\text{-}SLBP(def)}$ is the single-point energy of d-SLBP in the fully-optimized geometry of the adsorption complex after removing the gas molecule; $E_{d\text{-}SLBP}$ is the energy of an individual d-SLBP.

S3. Computational Results on Adsorption of Gas Molecules on Perfect SLBP

Relationships between E_{ad} and $d_{shortest}$ are shown in Figure S3 and Figure S4. All **1O*v*** or **O*v*** configurations for dioxides (NO_2, SO_2, CO_2) exhibit less negative E_{ad} values compared with other configurations at similar $d_{shortest}$, and thus are unfavorable to adsorption (Figure S3a–c). For example, E_{ad} values for **1O*v*** configurations of NO_2 range from –0.133 to –0.047 eV, whereas those for other configurations vary from –0.225 to –0.092 eV (Figure S3a). E_{ad} values of **S*va***, **S*vz***, ***pz***, ***pa*** and **2O*vz*** configurations of SO_2 are more negative than that of **1O*v*** or **2O*va*,** especially at short dshortest (Figure S3b). E_{ad} values for all ***pa*** or ***pz*** configurations of CO_2 are lower than those for **O*v*** vertical configurations (Figure S3c). ***Pa*** or ***pz*** configurations of SO_2 exhibit lower E_{ad} values than most other configurations (except for **S*vz***), especially at short $d_{shortest}$. The parallel configuration is not favored by NO_2 according to the E_{ad} values (Figure S3a).

Although NO (and CO) is a linear molecule similar to CO_2, parallel configurations are not energetically more favorable than vertical configurations until $d_{shortest}$ < 2.88 Å for NO (Figure S3d) or < 3.39 Å for CO (Figure S3e). For NH_3, nitrogen-pointing (**N*va*** or **N*vz***) configurations show slightly lower E_{ad} values than hydrogen-pointing configurations (**1H*v***, **3H*v***) (Figure S3f). The computational results prove that initial orientations cause remarkable impacts on favorable configurations and E_{ad} values for SLBP-adsorbing gas molecules.

For each gas molecule, impacts of initial adsorption sites on fully-optimized configurations and E_{ad} values are not as obvious as those of initial orientations (Figure S4). The most stable configurations of NO_2, NO, CO and NH_3 tend to be adsorbed at the H site. The most stable configuration is at the T or H site for SO_2, and B site for CO_2.

S4. The Most Stable Configuration of a Gas Molecule on SLBP and Related Disputes

The lowest-energy configuration obtained for NO_2 is an H type **2O*vz*** configuration (E_{ad} = –0.225 eV) at $d_{shortest}$ = 2.91 Å (Figure S3a). For **N*va*** or **N*vz***, the lowest-energy configuration is an H type **N*va*** configuration (E_{ad} = –0.211 eV, $d_{shortest}$ = 3.18 Å) or a T type **N*vz*** configuration (E_{ad} = –0.202 eV, $d_{shortest}$ = 2.80 Å), respectively (Figure S3a). Fully-optimized geometries of these

configurations are shown in Figure S4. Both the nitrogen-pointing configurations exhibit slightly less negative E_{ad} values than the H type ***2Ovz*** configuration (Figure S3a).

The most stable configuration for LBP adsorbing NO_2 has been a controversial issue. Cho et al. [4] suggest that two oxygen atoms pointing to LBP is the most stable adsorption configuration for NO_2, which is structurally close to the cycloaddition adsorption configuration between NO_2 and graphene [5]. The configuration of Cho et al. [4] is structurally similar to the H type ***2Ovz*** configuration (Figure S5a) in this study. The interaction energy calculated by Cho et al. [4] (–0.273 eV) is a bit lower than the E_{ad} value (–0.225 eV) calculated for H type ***2Ovz*** in this study. The small energy difference is most likely attributable to a small cutoff energy (408 eV) or a different size of box (not specified) used in that study.

Both Kou et al. [6] and Cai et al. [7] showed that the nitrogen-pointing configuration is the most favorable adsorption configuration for NO_2 on LBP. Their configurations are structurally similar to the H type ***Nva*** configuration (Figure S5b) and the T type ***Nvz*** configuration (Figure S5c) obtained in this study. However, remarkably short distances (2.2 or 2.27 Å) and high adsorption energies (–0.62, –0.5 eV) were reported in their studies, mainly due to employing a local density approximation (LDA) method [6] (the calculation method of Cai et al. [7] was not specified, but the calculation results are close to those of Kou et al. [6]). LDA is well known to underestimate bonding distance and overestimate binding energy in physisorbed systems [8]. According to the calculated E_{ad} values (Figure S3a), the H type ***2Ovz*** is a more favorable adsorption configuration than the H type ***Nva*** or the T type ***Nvz*** for NO_2 on SLBP.

Two configurations observed for SO_2 showed the lowest energies (Figure S3b), including a T type ***pz*** (E_{ad} = –0.310 eV, $d_{shortest}$ = 2.91 Å) and an H type ***Svz*** (E_{ad} = –0.309 eV, $d_{shortest}$ = 3.07 Å). Yang et al. [9] and Guo et al. [10] suggest that the most stable adsorption configuration for SO_2 is parallel to LBP at a distance of 2.59 or 2.84 Å, respectively. However, Kaewmaraya et al. [11] indicate that SO_2 prefers to adsorb on the H type site with sulfur pointing to LBP. This dispute over SO_2 is clarified in this study.

Configurations suggested by Yang et al. [9] and Guo et al. [10] are structurally similar to the T type ***pz*** configuration (Figure S5d) in this study. The distance calculated between SO_2 and SLBP in this study is close to that in the study of Guo et al. [10], but is 0.32 Å larger than that in the study of Yang et al. [9], where the LDA method was used. The configuration of Kaewmaraya et al. [11] is close to the H type ***Svz*** configuration (Figure S5e) in this study. The distance (3.00 Å) between SO_2 and LBP calculated in the study of Kaewmaraya et al. [11] based on projector-augmented wave Perdew–Burke–Ernzerhof method is close to that of H type ***Svz*** in this study, and the E_{ad} value (–0.33 eV) in that study is a bit lower.

Our results show that both the T type ***pz*** and the H type ***Svz*** are the most favorable adsorption configurations for SO_2 on LBP. Besides, a T type ***pa*** configuration at $d_{shortest}$ = 3.04 Å shows a slightly higher E_{ad} value (–0.301 eV) than the lowest value, and is

also favorable for the adsorption of SO_2 (Figure S5f). In the above ***pz*** or ***pa*** configuration, the distance between sulfur and phosphorus atoms is shorter than that between oxygen and phosphorus atoms. This means that the sulfur atom in SO_2 shows higher affinity to LBP than the two oxygen atoms. This is different from NO_2, for which oxygen atoms show higher affinity to LBP than nitrogen atom.

Three linear molecules, CO_2, NO and CO, prefer parallel configurations, as indicated by E_{ad} values (Figure S3c-S3e). The minimum E_{ad} value for CO_2 corresponds to a B type ***pa*** configuration at $d_{shortest}$ = 3.44 Å, where CO_2 is almost completely parallel to the SLBP plane with a negligible dihedral angle (Figure S5g). For NO or CO, the lowest-energy configuration is H type ***pa***, and the dihedral angle to the SLBP plane is 9°–17° (Figure S5h,i,j). This small dihedral angle is mainly attributed to asymmetry of monoxide molecules. The lowest-energy configuration for NH_3 is an H type **N*va*** (E_{ad} = –0.207 eV) at $d_{shortest}$ = 3.17 Å (Figure S3f). The nitrogen atom in both NO (Figure S5h) and NH_3 (Figure S5k) shows high affinity to SLBP, whereas oxygen atoms in NO_2 show high affinity to SLBP (Figure S5a).

S5. Computational Results on Individual d-SLBP

Fully-optimized geometries of SLBP and d-SLBP are shown in Figure 1. The horizontal and vertical P–P bonds of SLBP were calculated to be 2.22 and 2.26 Å in length, respectively (Figure 1a). The sum of bond angles for each phosphorus atom is 304°. Formation of new P–P bonds or breakage of original P–P bonds is indicated by the length between two phosphorus atoms in the fully relaxed geometries of d-SLBP (Figure 1b–k).

Thermodynamic stabilities of d-SLBP are evaluated by calculating E_f defined as Equation (6) [12] or (7):

$$E_f\ (\text{in-plane defect}) = E_{d\text{-SLBP}} - m \times E_P \quad (6)$$

$$E_f\ (\text{edge defect}) = E_{d\text{-SLBP}} - m \times E_P - k \times \mu_H \quad (7)$$

where $E_{d\text{-SLBP}}$ represents the vdW force-corrected energy of d-SLBP; E_P is the energy of each phosphorus atom in SLBP; μ_H is chemical potential of one hydrogen atom in an H_2 molecule; m is the number of phosphorus atoms in d-SLBP; k is the number of hydrogen atoms added to saturate phosphorus atoms on the edge.

E_f values calculated for d-SLBP range from 1.24 to 4.98 eV (Table S1). A low E_f value indicates a stable defect [13]. SW defects have the lowest E_f values (1.24, 1.52 eV) and are the most stable defects in LBP. This is because no bond breakage or formation occurs while forming SW defects. Formation of SW involves only rotation of P–P bonds. SV and DV2 have the second lowest E_f values (1.77, 1.71 eV), because new bond formation releases energy and compensates partially for the bond breaking energy while forming a defect. DV1 and DV3, together with EGa1 and EGa2, are among the defects possessing the largest E_f values (3.58–4.94 eV). EGz1 and EGz2 show E_f values (2.63, 2.82 eV) lower than those of EGa1 and EGa2 (4.77, 4.94 eV). This means

that edge defects formed in the zigzag configuration are much more stable than those formed in the armchair configuration, reflecting the anisotropy of LBP.

Table S1. Formation energies (E_f, eV) of defects in LBP and graphene.

SLBP	E_f (This study)	E_f (LBP [12])	E_f (Graphene [14])
Perfect	0	0	0
SW1	1.24	1.02 – 1.32	4.50 – 5.30
SW2	1.52		
SV	1.77	1.63 – 2.03	7.38 – 7.85
DV1	3.58	1.91 – 3.04	7.52 – 8.70
DV2	1.71		
DV3	4.43	-[a]	6.40 – 7.50
EGz1	2.63	-[a]	-[a]
EGz2	2.82	-[a]	-[a]
EGa1	4.77	-[a]	-[a]
EGa2	4.94	-[a]	-[a]

a. No available data.

E_f values of d-SLBPs are systematically lower than those of defective graphene (4.5–8.7 eV [14]), meaning defects are easier to form in SLBP than in graphene (Table S1). E_f values for SW, SV or DV2 calculated in this study are close to the formation energies of similar defects in LBP calculated by Hu et al. [12]. E_f values of DV1 and DV3 are higher than those previously reported [12].

DV1 in this study is structurally similar to the DV-(5|8|5)-1 in the study of Hu et al. [12]. Hu et al. constructed one DV-(5|8|5)-1 in a 5 × 1 × 7 supercell, which is larger than twice the size of the box used in this study. If considering the two periodic boxes used in this study, there will be one DV1 defect neighbor to the current DV1 defect in each of the ***a*** and ***c*** directions. As all DV1 defects are equal, phosphorus atoms between two neighboring defects are not able to move toward any of the defect centers. As a result, single electron orbitals of unsaturated phosphorus atoms in a defect center are not able to overlap. In contrast, phosphorus atoms of an isolated defect in a large supercell are capable of moving slightly toward the defect center, causing a large amount of orbital overlap, and releasing energy to compensate for the bond breaking energy. Therefore, the E_f value for the same defect calculated in this study is higher than that in the study of Hu et al.

DV3 is constructed by removing two unbonded phosphorus atoms from two neighboring honeycomb structures in this study (Figure S9g). This is different from the DV-(555|777) defect constructed by Hu et al. [12]. DV-(555|777) is constructed by removing two phosphorus atoms in the same honeycomb structure. From a geometry aspect, DV3 in this study could be regarded as two SV defects. As the newly formed P_{13}–P_{20} single bond in SV lowers the E_f value, the E_f value (4.43 eV) of DV3 is slightly higher than the two E_f values of SV (1.77 eV).

To identify the locations of unpaired single electrons for each d-SLBP, Mulliken population analysis was performed to characterize the spin density in each atom. No atoms carry dangling single electrons in SW1 or SW2, as indicated by zero spin

density (Figure 3a,b), because no phosphorus atoms are removed in construction, albeit the geometry deformation induced by the defect is significant (Figure 1b,c). The distance between unsaturated atoms P_{13} and P_{17}, or P_{15} and P_{19} (2.94 Å), in the fully-optimized geometry of DV1 (Figure 1e) is a bit larger than the typical length of a P–P single bond (2.2 Å). Spin densities distributed on the four unsaturated atoms are less than ±0.02 (Figure 3d), thereby not enhancing the interaction with NO_2. New bonds P_7–P_{22} (2.40 Å) and P_{13}–P_{20} (2.40 Å) are formed in the fully relaxed geometry of DV2 (Figure 1f). Zero spin density in DV2 (Figure 3e) confirms that there are no unsaturated atoms. Non-zero spin densities in DV3 concentrate mainly on unsaturated atoms P_{13}, P_{17}, P_{30} and P_{34}. Dangling single electrons on atoms P_{19} and P_{20} pair with each other, resulting in zero spin density distribution around the two phosphorus atoms (Figure 3f).

Almost no deformation is observed in the fully relaxed geometry of EGz1 including three inherently unsaturated atoms, $\mathbf{P}_{27}$, $\mathbf{P}_{31}$ and $\mathbf{P}_{35}$, on the zigzag edge (Figure 1h). The three phosphorus atoms exhibit the most concentrated spin densities (Figure 3g) and are major locations for single electrons. The fully-relaxed geometry of EGz2 shows slight deformation compared with that of EGz1 due to the absence of $\mathbf{P}_{31}$ (Figure 1i). Zero spin densities are present for EGz2 (Figure 3h), indicating that single electrons on unsaturated atoms $\mathbf{P}_{27}$, $\mathbf{P}_{32}$, $\mathbf{P}_{35}$ and $\mathbf{P}_{36}$ pair with each other. In the fully relaxed geometry of EGa1, the original bond $\mathbf{P}_{17}$–$\mathbf{P}_{22}$ (2.22 Å) is broken, and the distance between the two phosphorus atoms is elongated to 3.39 Å (Figure 1j). Spin densities on eight unsaturated phosphorus atoms are zero (Figure 3i), indicating that there are no unpaired single electrons in EGa1. Removal of $\mathbf{P}_{21}$ for constructing EGa2 leads to the formation of a new bond, $\mathbf{P}_{11}$–$\mathbf{P}_{22}$ (2.23 Å), after geometry relaxation (Figure 1k). The vast majority of spin densities (0.75) are concentrated on $\mathbf{P}_{11}$ (Figure 3j), which is regarded as a main location for single electrons in EGa2.

S6. Computational Results on Adsorption of Gas Molecules on d-SLBP

E_{ad} values calculated for all configurations on d-SLBP and most stable adsorption configurations for NO_2, SO_2 and CO_2 are shown in Table S2 and Figure S17–S19. Table S2 shows the E_{ad}, E_{vdW}/E_{ad} and $d_{shortest}$ of the most stable adsorption configuration of the three gases on each d-SLBP. SV, DV3, EGz1 and EGa2 significantly enhance the adsorption of NO_2, as indicated by the low E_{ad} values (–2.584 to –1.304 eV) in the most stable adsorption configurations. vdW force accounts for only 2%–14% of the total E_{ad} when E_{ad} is lower than –1 eV, implying typical chemical adsorption. In the most stable configuration for each of the four unsaturated d-SLBP, NO_2 combines with $\mathbf{P}_{17}$ in SV, $\mathbf{P}_{17}$ in DV3, $\mathbf{P}_{31}$ in EGz1 and $\mathbf{P}_{11}$ in EGa2 to form new $\mathbf{P}_{17}$–**O**, $\mathbf{P}_{31}$–**O** and $\mathbf{P}_{11}$–**O** (Figure S17). $\mathbf{P}_{17}$, $\mathbf{P}_{11}$ and $\mathbf{P}_{31}$ are the atoms with the largest spin densities in the d-SLBP. Therefore, NO_2 was found to bind with a P atom possessing the largest spin density, i.e., the location of a dangling single electron. For d-SLBP exhibiting zero or close to zero spin density, adsorption of NO_2 is not enhanced. For SO_2 and CO_2, only E_{ad} on DV3 is significantly more negative. $d_{shortest}$ values between the two gas molecules and d-SLBP not including DV3 are comparable to those for absorption on SLBP (Figures S18 and S19).

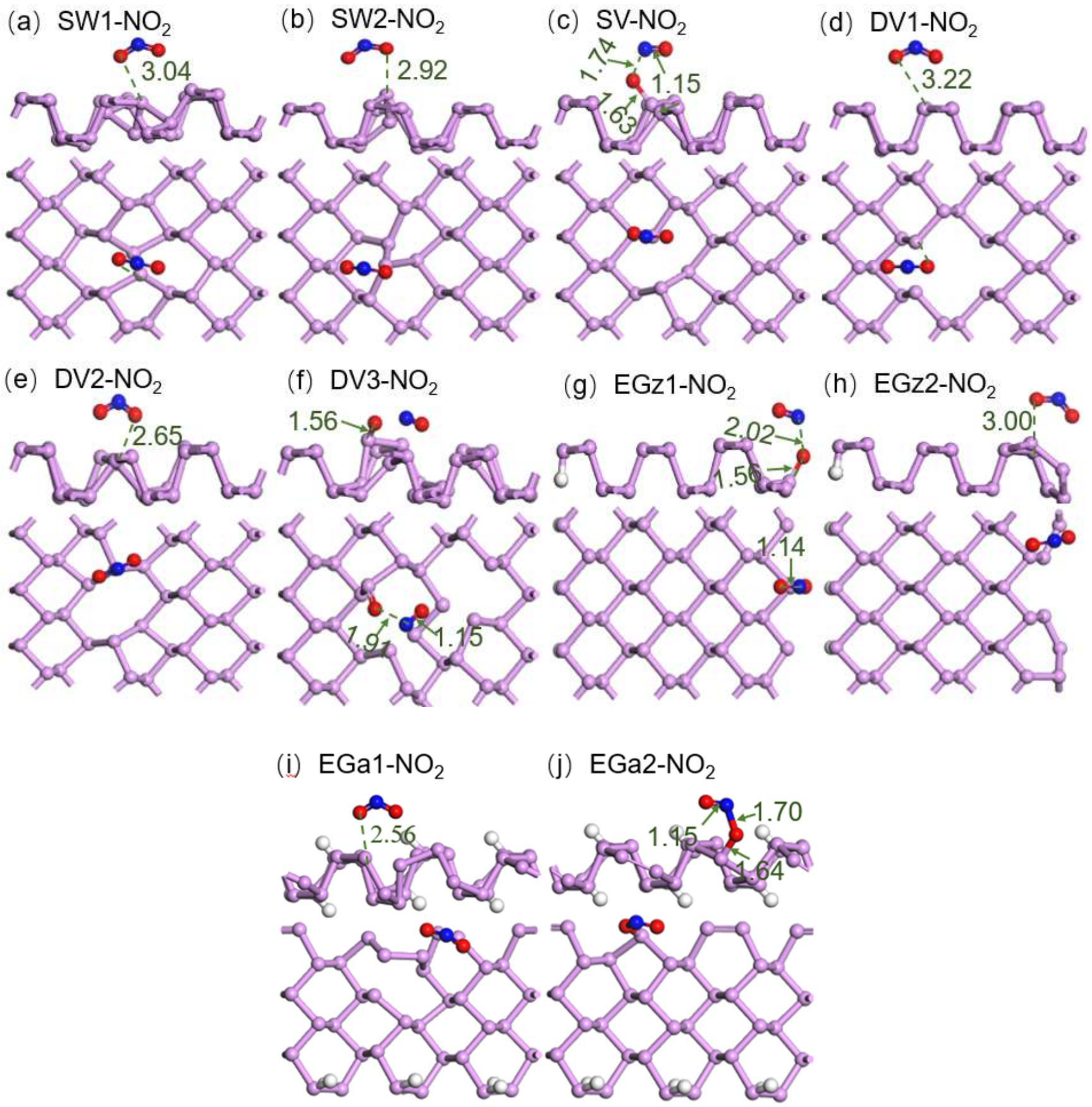

Figure S17. Side and top views of the most energetically stable adsorption configurations calculated for NO_2 and d−SLBP. (a) SW1−NO_2, (b) SW2−NO_2, (c) SV−NO_2, (d) DV1−NO_2, (e) DV2−NO_2, (f) DV3−NO_2, (g) EGz1−NO_2, (h) EGz2−NO_2, (i) EGa1−NO_2 and (j) EGa2−NO_2. Green numbers: the shortest atom distance ($d_{shortest}$) between d−SLBP and NO_2. Pink balls: phosphorus atoms; red balls: oxygen atoms; blue balls: nitrogen atoms; light grey balls: hydrogen atoms.

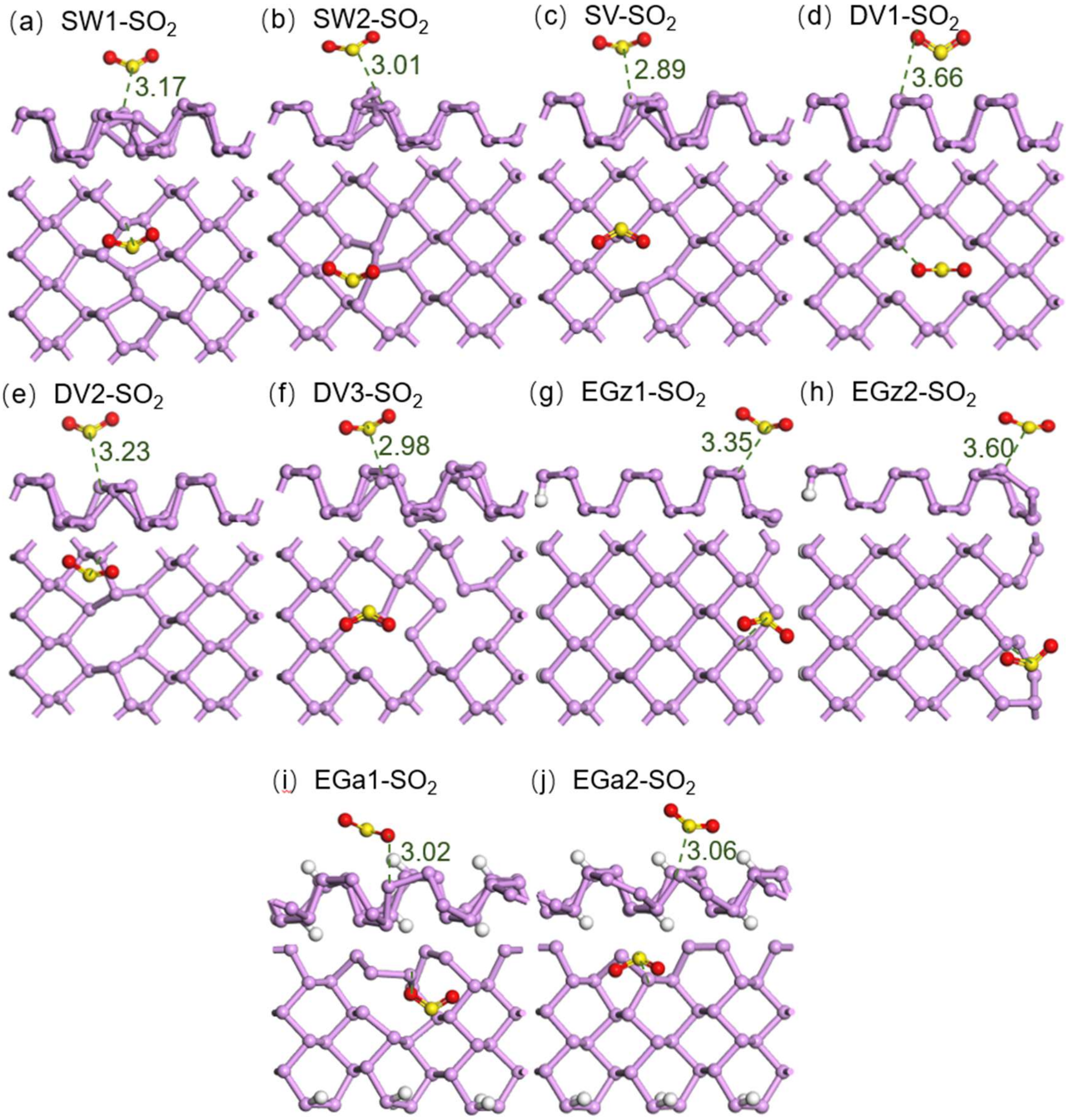

Figure S18. Side and top views of the most energetically stable adsorption configurations of SO_2 on d−SLBP. (a) SW1−SO_2, (b) SW2−SO_2, (c) SV−SO_2, (d) DV1−SO_2, (e) DV2−SO_2, (f) DV3−SO_2, (g) EGz1−SO_2, (h) EGz2−SO_2, (i) EGa1−SO_2 and (j) EGa2−SO_2. Green numbers represent the shortest atom distances ($d_{shortest}$) between d−SLBP and SO_2.

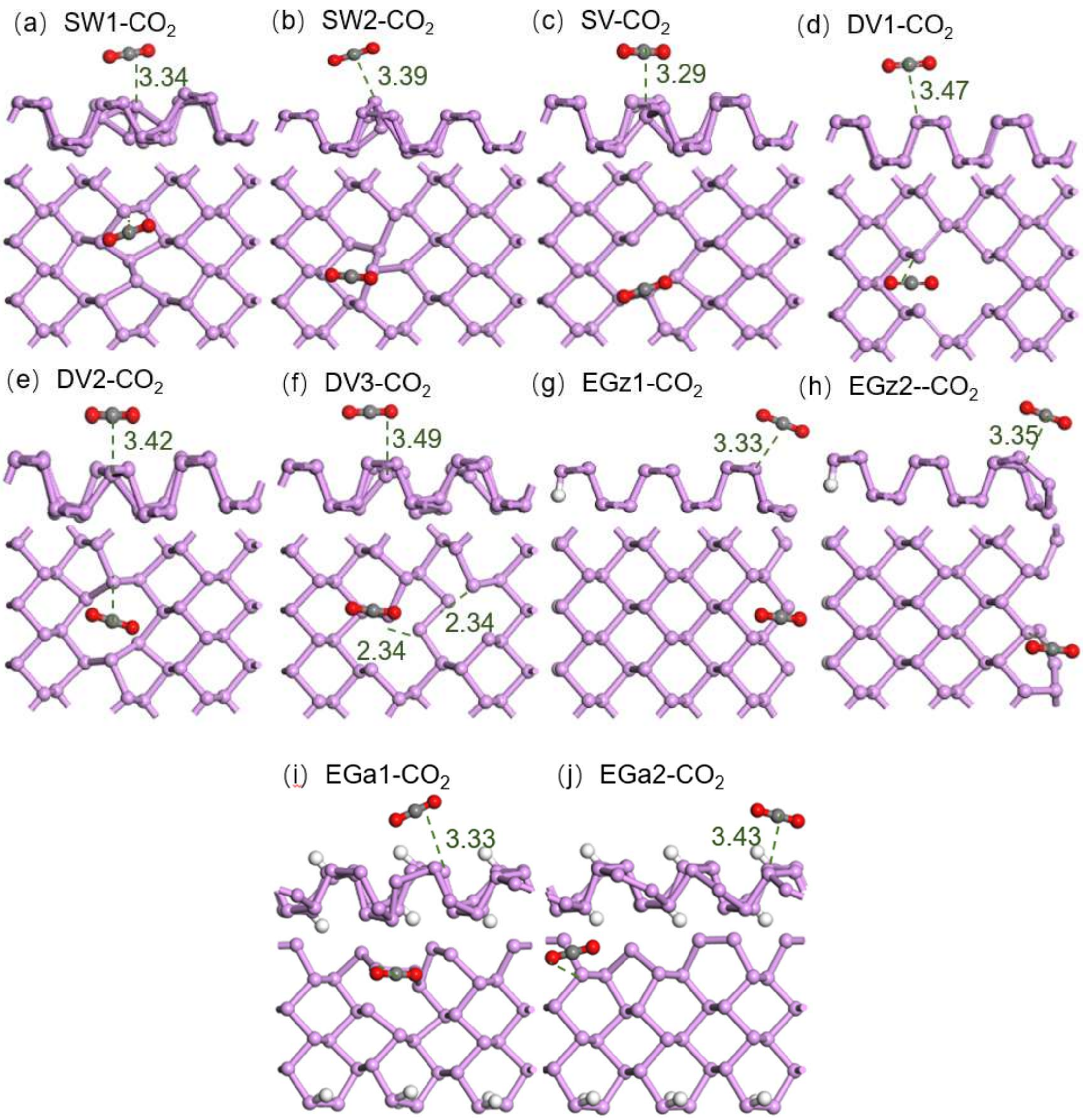

Figure S19. Side and top views of the most energetically stable adsorption configurations of CO_2 on d−SLBP. (a) SW1−CO_2, (b) SW2−CO_2, (c) SV−CO_2, (d) DV1−CO_2, (e) DV2−CO_2, (f) DV3−CO_2, (g) EGz1−CO_2, (h) EGz2−CO_2, (i) EGa1−CO_2 and (j) EGa2−CO_2. Green numbers represent the shortest atom distances ($d_{shortest}$) between d−SLBP and CO_2.

Table S2. Adsorption energies (E_{ad}, eV), E_{vdW}/E_{ad} (%), the shortest distances ($d_{shortest}$, Å) and charge transfer (Δq, e) of the lowest-energy adsorption configurations of NO_2, SO_2 and CO_2 on SLBP and each d-SLBP.

BP	NO_2				SO_2				CO_2			
	E_{ad}	E_{vdW}/E_{ad}	$d_{shortest}$	Δq	E_{ad}	E_{vdW}/E_{ad}	$d_{shortest}$	Δq	E_{ad}	E_{vdW}/E_{ad}	$d_{shortest}$	Δq
SLBP	-0.225	71%	2.91	-0.14	-0.310	63%	2.91	-0.11	-0.156	79%	3.44	-0.01
SW1	-0.198	78%	3.04	-0.11	-0.256	69%	3.17	-0.06	-0.144	103%	3.34	-0.01
SW2	-0.154	65%	2.92	-0.13	-0.193	53%	3.03	-0.06	-0.098	85%	3.39	0
SV	-1.433	13%	1.63	-[a]	-0.289	56%	2.89	-0.10	-0.144	102%	3.29	-0.01
DV1	-0.198	65%	3.22	-0.06	-0.214	89%	3.66	0.01	-0.137	93%	3.47	0
DV2	-0.240	65%	2.65	-0.22	-0.256	57%	3.23	-0.05	-0.162	100%	3.42	-0.01
DV3	-2.584	2.6%	1.56	-[a]	-1.378	4%	2.98	-0.10	-1.211	-1.7%	3.49	0
EGz1	-1.304	14%	1.56	-[a]	-0.177	64%	3.34	-0.04	-0.132	77%	3.33	-0.02
EGz2	-0.229	58%	3.00	-0.11	-0.165	65%	3.60	-0.01	-0.140	81%	3.35	-0.01
EGa1	-0.255	59%	2.56	-0.29	-0.318	69%	3.02	-0.10	-0.127	90%	3.35	-0.02
EGa2	-1.543	2%	1.64	-[a]	-0.200	53%	3.06	-0.07	-0.114	99%	3.42	0

a. NO_2 dissociates and transforms into an [**NO**] species. The nature of [**NO**] is discussed in S7.

S7. Nature of the [NO] Species.

The strong interaction between SV and NO_2 causes **N**–**O** bond cleavage to form **[NO]** species, and simultaneous oxidation of the unsaturated $\mathbf{P}_{17}$ in SV. To know the nature of **[NO]**, we pulled it away from the main part of SV to construct a desorption system preventing any interaction between the two parts. The desorption configuration after geometry relaxation is shown in Figure S20. The Mulliken atomic population of the desorption configuration was analyzed (Table S3). Total charge distributed on [**NO**] is zero, and the total spin population is 1. This means that [**NO**] is a nitrogen monoxide molecule in nature.

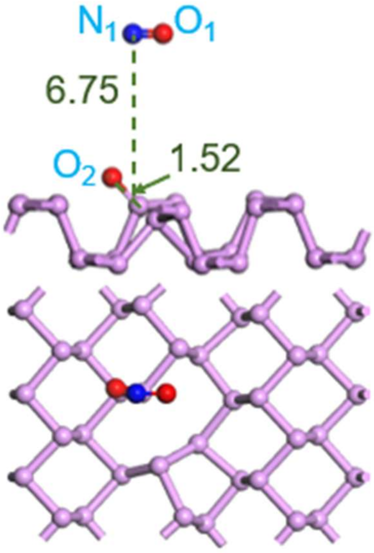

Figure S20. Side and top views of the desorption configuration.

Table S3. Charge and spin population on each atom in the desorption configuration of SV absorbing NO_2 by Mulliken atomic populations.

Atom	Total electrons	charge(e)	Spin(hbar/2)
N_1	4.84	0.16	0.70
O_1	6.16	-0.16	0.30
O_2	6.88	-0.88	-0.19
P_1	5.01	-0.01	0.00
P_2	4.99	0.01	-0.01
P_3	4.99	0.01	-0.02
P_4	5.00	-0.00	-0.00
P_5	5.00	-0.00	0.00
P_6	4.96	0.04	0.00
P_7	5.05	-0.05	-0.07
P_8	4.99	0.01	-0.00
P_9	5.01	-0.01	-0.01
P_{10}	4.93	0.07	-0.06
P_{11}	4.99	0.01	-0.00
P_{12}	4.99	0.01	-0.08
P_{13}	5.02	-0.02	-0.06
P_{14}	5.00	-0.00	-0.04
P_{15}	4.98	0.02	-0.00
P_{16}	5.00	0.00	-0.01
P_{17}	4.17	0.83	-0.29
P_{19}	5.01	-0.01	-0.01
P_{20}	5.02	-0.02	-0.00
P_{21}	4.98	0.02	-0.03
P_{22}	5.05	-0.05	-0.05
P_{23}	5.00	0.00	-0.03
P_{24}	5.00	0.00	0.00
P_{25}	5.01	-0.01	-0.00
P_{26}	5.00	0.00	-0.00
P_{27}	5.00	0.00	-0.00
P_{28}	5.01	-0.01	-0.00
P_{29}	4.99	0.01	-0.00
P_{30}	5.00	0.00	-0.00
P_{31}	4.99	0.01	-0.01
P_{32}	5.00	0.00	0.00
P_{33}	4.99	0.01	-0.00
P_{34}	5.00	-0.00	-0.01
P_{35}	5.00	0.00	-0.01
P_{36}	5.00	0.00	-0.00

S8. Deformation of DV3 after interacting with gas molecules

Large E_{ad} values of dioxidieds interacting with DV3 are attributed to the deformation of the defect center. DV3 is severely deformed when a gas molecule is adsorbed. Distances of $\mathbf{P}_{17}$ and $\mathbf{P}_{20}$, and $\mathbf{P}_{19}$ and $\mathbf{P}_{34}$ are reduced from 3.52 Å to 2.34 Å to form new bonds $\mathbf{P}_{17}$–$\mathbf{P}_{20}$ and $\mathbf{P}_{19}$–$\mathbf{P}_{34}$ (Figure S21). The deformed DV3 is structurally similar to those of two independent single-vacancy defects. Table S4 summarizes the deformation energies (E_{def}) of all d-SLBP after adsorbing gas molecules. E_{def} for d-SLBP interacting with NO_2 was calculated to be –0.718 to 0.254 eV (Table S4). After deducing E_{def} from E_{ad}, the modified interaction

energy ($E_{ad\text{-}def}$) for NO_2 interacting with DV3 is –1.866 eV, which is comparable to those calculated for SV, EGz1 or EGa2 (–1.543 to –1.304 eV) (Figure S6).

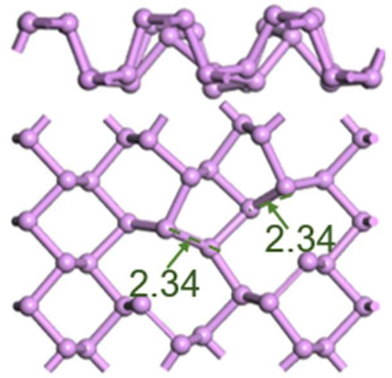

Figure S21. Side and top views of deformed DV3 after adsorption.

Table S4 Interaction energies (E_{ad}, eV), deformation energies (E_{def}, eV) and modified interaction energies ($E_{ad\text{-}def}$, eV) for d-SLBP and dioxides.

BP	NO_2			SO_2			CO_2		
	E_{ad}	E_{def}	$E_{ad\text{-}def}$	E_{ad}	E_{def}	$E_{ad\text{-}def}$	E_{ad}	E_{def}	$E_{ad\text{-}def}$
SW1	-0.198	0.004	-0.202	-0.256	0.003	-0.259	-0.144	0.002	-0.146
SW2	-0.154	0.034	-0.188	-0.193	0.030	-0.224	-0.098	-0.002	-0.096
SV	-1.433	0.188	-1.620	-0.289	0.009	-0.298	-0.144	0.013	-0.157
DV1	-0.198	0.000	-0.198	-0.214	-0.006	-0.208	-0.137	0.007	-0.144
DV2	-0.240	0.024	-0.264	-0.256	0.006	-0.262	-0.162	0.009	-0.171
DV3	-2.584	-0.718	-1.866	-1.378	-1.076	-0.302	-1.211	-1.074	-0.137
EGz1	-1.304	0.218	-1.523	-0.177	-0.001	-0.176	-0.132	-0.020	-0.113
EGz2	-0.229	-0.013	-0.216	-0.165	-0.019	-0.147	-0.140	-0.023	-0.116
EGa1	-0.255	0.033	-0.288	-0.318	0.008	-0.326	-0.127	-0.001	-0.126
EGa2	-1.543	0.254	-1.691	-0.200	-0.009	-0.201	-0.114	-0.010	-0.094

S9. Single-Electron-Dominated Mechanism for SLBP or d-SLBP Absorbing Gas Molecules.

Total (TDOS) and partial DOS (PDOS) calculation results for SLBP or d-SLBP before absorbing gas molecules are shown in Figure S22. The results show that SLBP is a direct semiconductor possessing a bandgap of 0.91 eV (Figure S22a). This bandgap value is close to the lower range of experimental values (1.0–1.5 eV) [15], and in accordance with most GGA-PBE based calculation results (0.86–0.91 eV) [16].

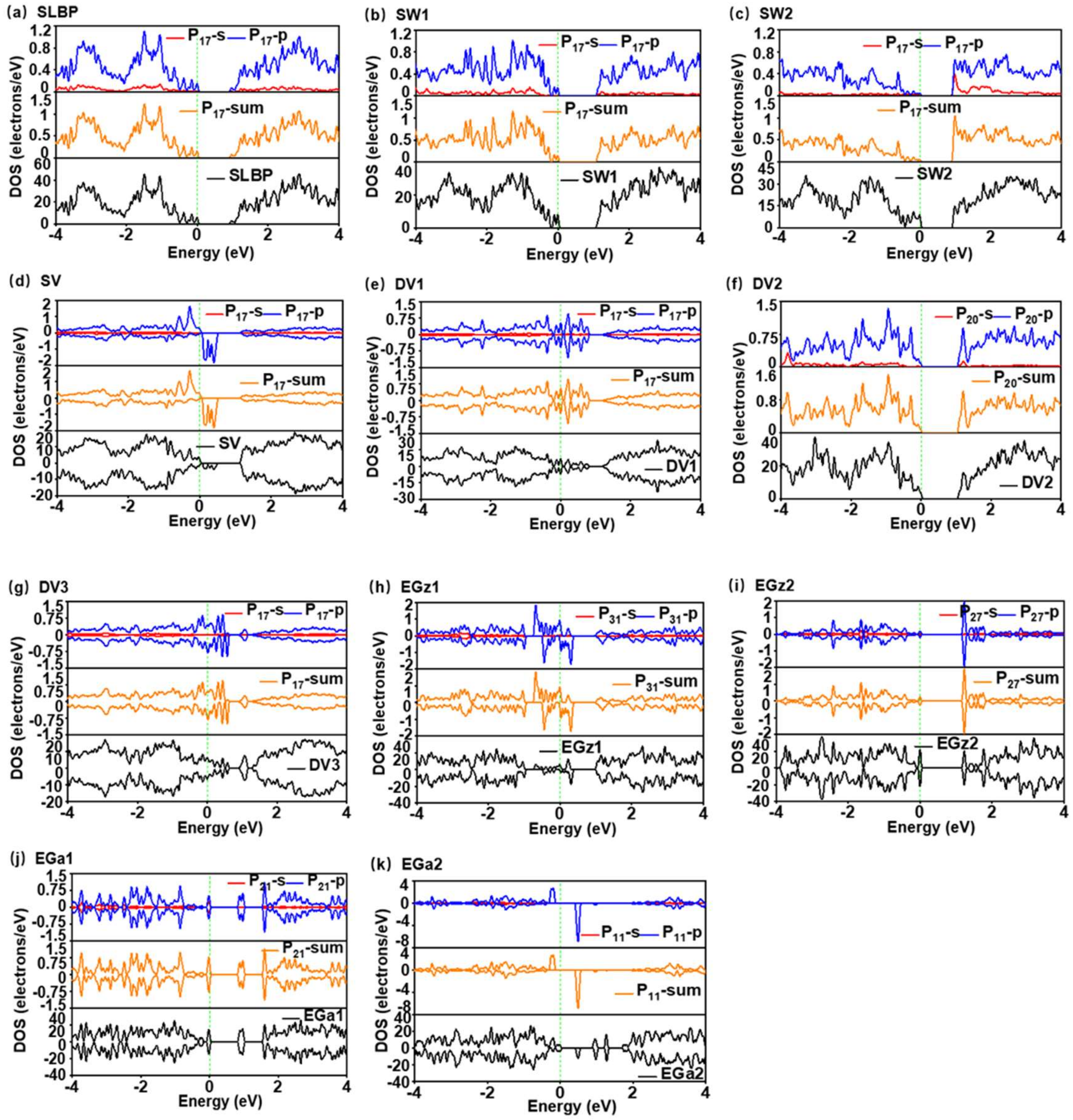

Figure S22. Total density of states (TDOS) of SLBP and d−SLBP and partial DOS (PDOS) of key phosphorus atoms contributing to changes of total DOS. (a) Atom $\mathbf{P}_{17}$ in SLBP, (b) atom $\mathbf{P}_{17}$ in SW1, (c) atom $\mathbf{P}_{17}$ in SW2, (d) atom $\mathbf{P}_{17}$ in SV, (e) atom $\mathbf{P}_{17}$ in DV1, (f) atom $\mathbf{P}_{20}$ in DV2, (g) atom $\mathbf{P}_{17}$ in DV3, (h) atom $\mathbf{P}_{31}$ in EGz1, (i) atom $\mathbf{P}_{27}$ in EGz2, (j) atom $\mathbf{P}_{21}$ in EGa1 and (k) atom $\mathbf{P}_{11}$ in EGa2. Green dashed line: Fermi level.

Introducing defect SW1, SW2 or DV2 carrying no unpaired electrons cannot change semiconducting nature, albeit causing a slight impact on electronic structure and bandgap (Figure S22b,c,f). In DOS of other defects, new states appear near or above the Fermi level (Figure S22d,e,g–k). TDOS of DV1, EGz2 or EGa1 is spin symmetric, in accordance with zero or negligible spin populations. Atoms $\mathbf{P}_{17}$ in DV1, $\mathbf{P}_{27}$ in EGz2 and $\mathbf{P}_{21}$ in EGa1 were selected as representatives to examine PDOS. The PDOS is symmetric (Figure S22e,i,j), further confirming that all orbitals are occupied by electrons and no atoms carry unpaired single electrons in DV1, EGz2 or EGa1.

TDOS of SV, EGz1 and EGa2 are obviously spin-asymmetric (Figure S22d,h,k). As shown in PDOS, the spin asymmetry is mainly contributed by the spin-asymmetric *p* orbital of the unsaturated atom $\mathbf{P}_{17}$ in SV, $\mathbf{P}_{31}$ in EGz1 or $\mathbf{P}_{11}$ in EGa2. This is owed to the presence of a single electron in the *p* orbital according to the non-zero spin population. DV3 is special due to possessing symmetric TDOS but asymmetric PDOS, taking atom $\mathbf{P}_{17}$ as an example (Figure S22g). This is attributed to the non-zero spin population on atom $\mathbf{P}_{17}$. Similarly, *p* orbitals of the other three unsaturated atoms $\mathbf{P}_{13}$, $\mathbf{P}_{30}$ and $\mathbf{P}_{34}$ are spin-asymmetric. However, as spin-up offsets spin-down, no asymmetry can be observed in TDOS of DV3. The four single-electron d-SLBP all have spin-asymmetric TDOS or PDOS.

For SLBP, flat bands occur near the Fermi level and spin polarization is generated after adsorbing an NO_2 or NO containing a single electron (Figure S23a,d). According to DOS patterns, the flat band and spin-polarized electron is mainly located in NO_2 or NO. For NO_2, a flat band with spin-down electron appears above the Fermi level. Although NO_2 produces a doping state, the orbital overlap between NO_2 and SLBP is not as much as that of SO_2 and SLBP. The orbital overlap of SO_2 and SLBP is the largest among the six gas molecules (Figure S23b), which is consistent with its most negative E_{ad} value. SLBP retains semiconductor characteristics as a direct bandgap after interacting with SO_2, CO_2, CO or NH_3 (E_g = 0.907–0.946 eV). SO_2 induces some states in the conduction band at around 1 eV. Adsorption of CO_2, CO or NH_3 to SLBP produces no noticeable change to DOS near the Fermi level (Figure S23c,e,f), which manifests the weak vdW force between SLBP and gas.

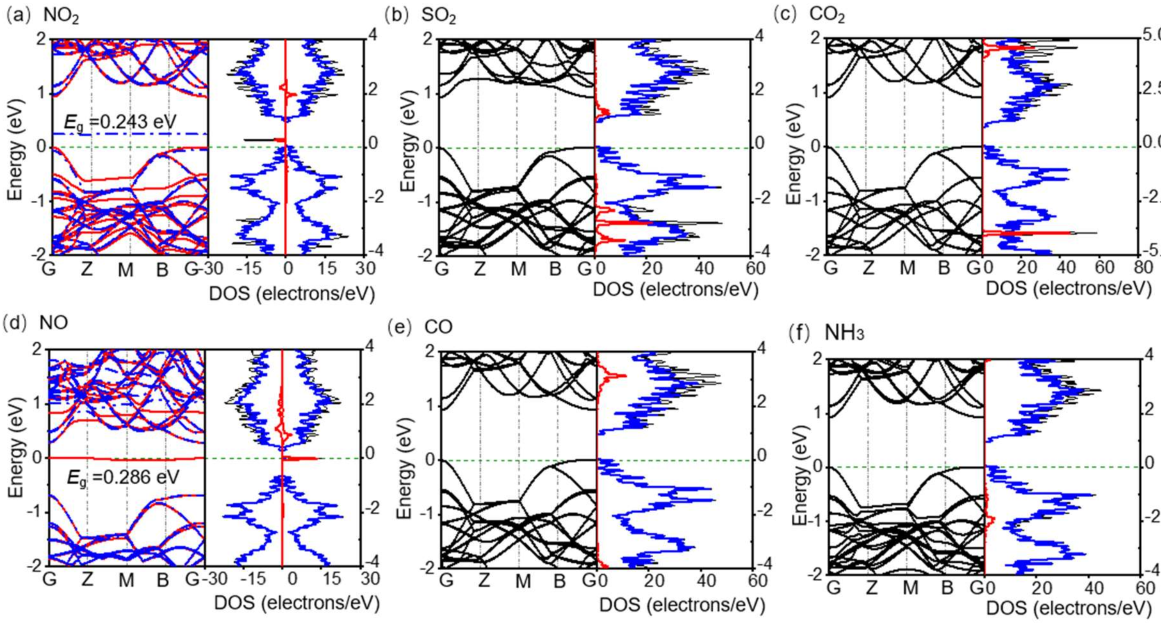

Figure S23. Band structures and DOS patterns of SLBP and gas adsorption complexes. (a) NO_2, (b) SO_2, (c) CO_2, (d) NO, (e) CO, (f) NH_3. In each band structure pattern, a red solid line and blue dashed line are spin-up and spin-down for NO_2 and NO; other bands are represented by black solid lines. In a DOS pattern, a black solid line is TDOS; a blue solid line is DOS of SLBP in an adsorption complex; a red solid line is the DOS of the gas molecule in the adsorption complex.

TDOS and PDOS of adsorption complexes of d-SLBP are shown in Figures S24–S26. Since NO_2 is a paramagnetic single-electron gas, the introduction of NO_2 causes asymmetric spin-up and spin-down polarization in all the d-SLBP except for EGa2. This asymmetry can be divided into two categories. One is SW1, SW2, DV1, DV2, EGz2 and EGa1; and the other is SV, DV3 and EGz1 (Figure S24). For defects of the first type, they do not contain single electrons. Since there is no single electron that can interact with these NO_2, the whole system exhibits spin asymmetry, which is mainly derived from the nitrogen and oxygen of NO_2.

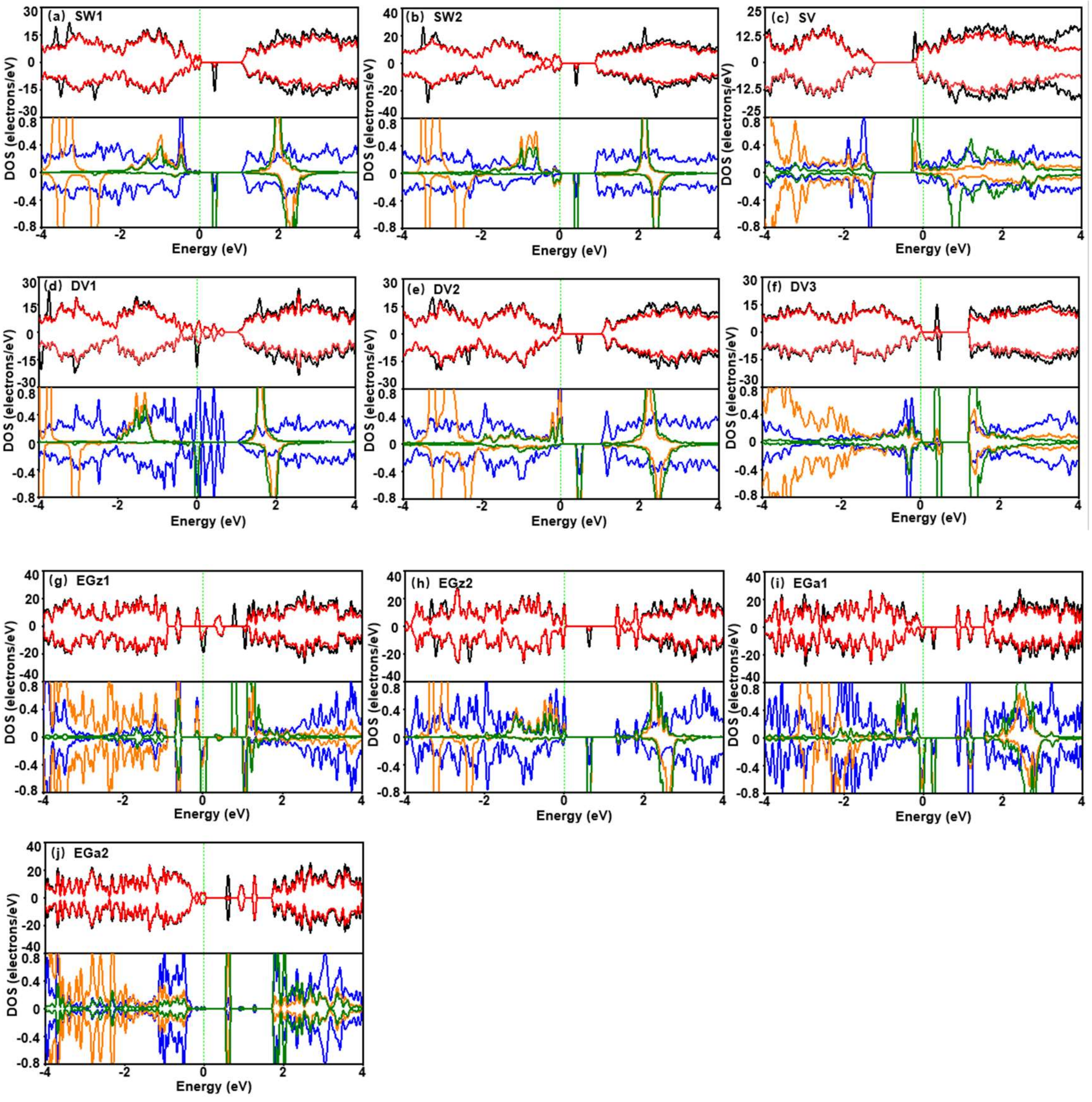

Figure S24. TDOS of adsorption complexes of d−SLBP and NO_2 (black solid line) and PDOS of d−SLBP (red solid line) in adsorption complexes (upper panel). (a) SW1, (b) SW2, (c) SV, (d) DV1, (e) DV2, (f) DV3, (g) EGz1, (h) EGz2, (i) EGa1 and (j) EGa2. PDOS of *p* orbitals of nitrogen (orange solid line) and oxygen (the one closer to adsorbate, dark green solid line) atoms and the phosphorus atom (blue solid line) nearest to NO_2 (lower panel). Green dashed line: Fermi level.

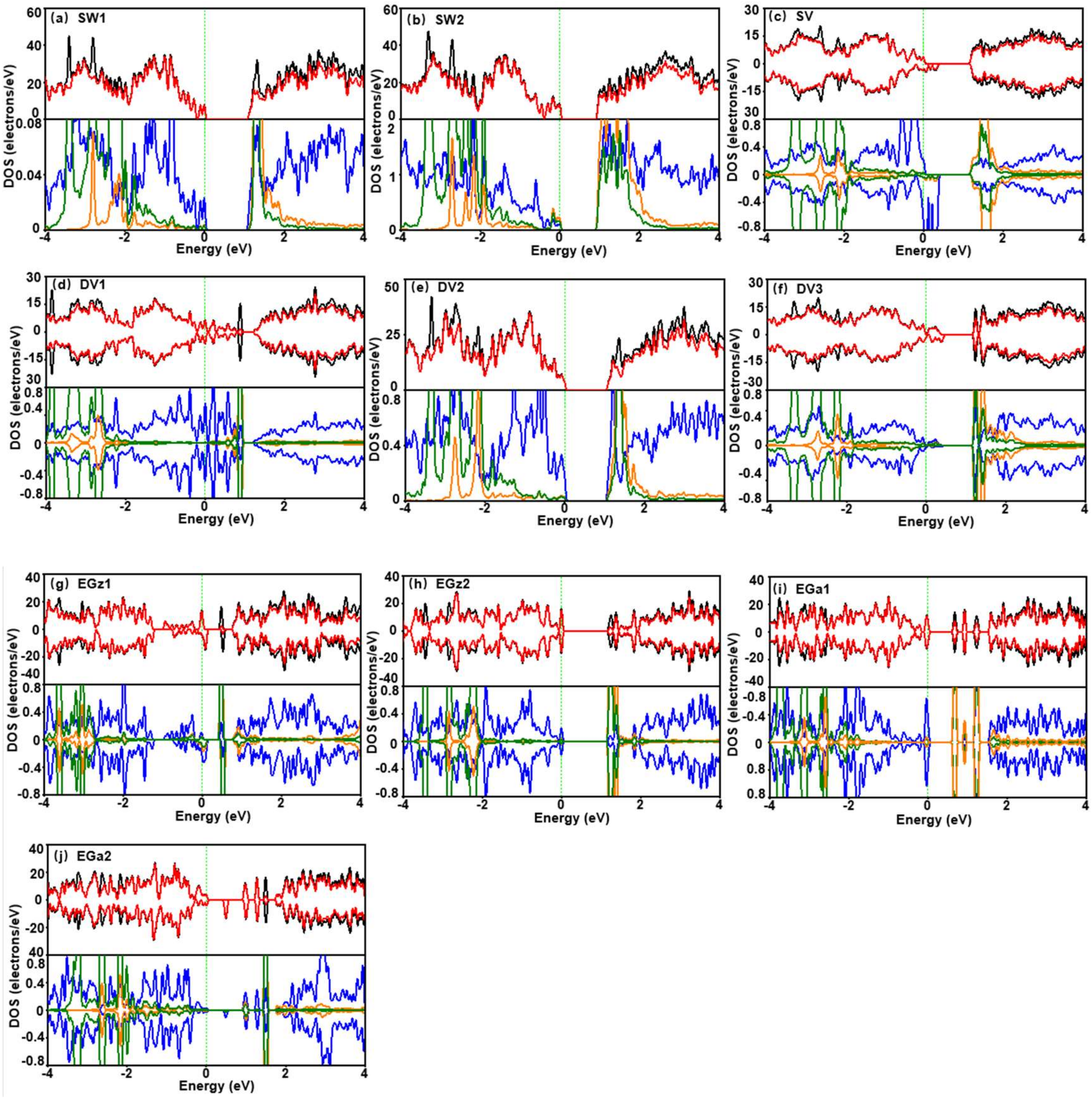

Figure S25. TDOS of adsorption complexes of d-SLBP and SO_2 (black solid line) and PDOS of d-SLBP (red solid line) in adsorption complexes (upper panel). (a) SW1, (b) SW2, (c) SV, (d) DV1, (e) DV2, (f) DV3, (g) EGz1, (h) EGz2, (i) EGa1 and (j) EGa2. PDOS of *p* orbitals of sulfur (orange solid line) and oxygen (the one closer to adsorbate, dark green solid line) atoms and the phosphorus atom (blue solid line) nearest to SO_2 (lower panel). Green dashed line: Fermi level.

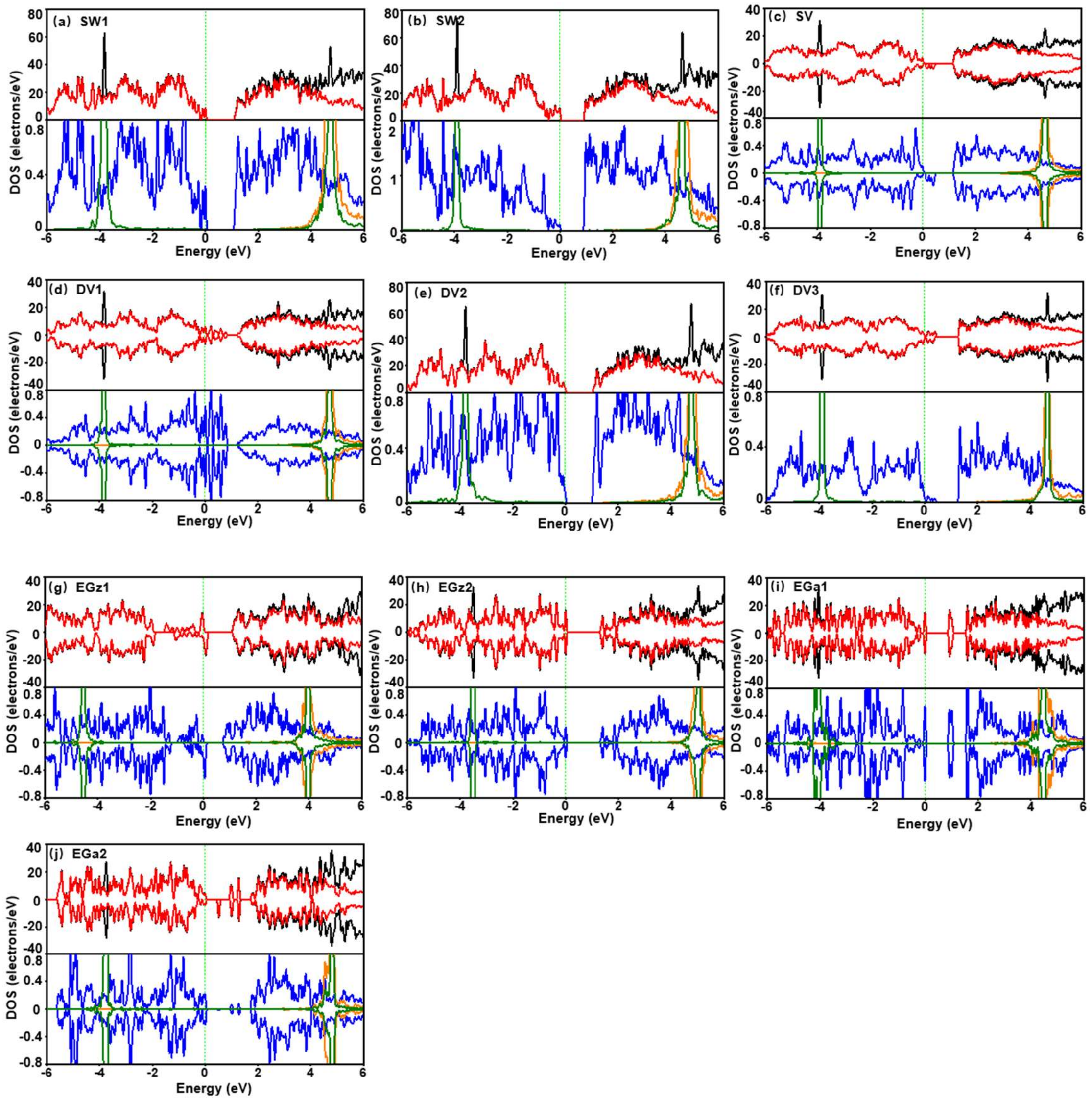

Figure S26. TDOS of adsorption complexes of CO_2 and d-SLBP (black solid line) and PDOS of d-SLBP (red solid line) in adsorption complexes (upper panel). (a) SW1, (b) SW2, (c) SV, (d) DV1, (e) DV2, (f) DV3, (g) EGz1, (h) EGz2, (i) EGa1 and (j) EGa2. PDOS of *p* orbitals of carbon (orange solid line) and oxygen (the one closer to adsorbate, dark green solid line) atoms and the phosphorus atom (blue solid line) nearest to CO_2 (lower panel). Green dashed line: Fermi level.

For the defects of the second type, individual SV or EGz1 own an odd number of single electrons, as shown by symmetric TDOS (Figure S22d,h), and DV3 owns an even number of single electrons, as shown by symmetric TDOS (Figure S22g). The single electrons can interact strongly with the single electrons in NO_2. This strong interaction causes the dissociation of NO_2 to form NO, which can be regarded as a system of single-electron NO and oxidation-d-SLBP. This asymmetry stems not only from nitrogen and oxygen, but also from the unsaturated $\mathbf{P}_{17}$, P_{17} and P_{31} in SV, DV3 and EGz1. For EGa2, it also contains a single electron (Figure S22k) that interacts strongly with NO_2, but it does not dissociate from NO_2. According to the lowest energy and Pauli exclusion

principle [23], the single electrons of EGa2 and undissociated NO_2 tend to pair with each other to form a stable structure, thereby exhibiting a distinct spin symmetry.

Since there is no single electron in SO_2 or CO_2, the original spin characteristics of the d-SLBP are still maintained after combination (Figures S25 and S26), and no major changes in the electronic properties of the d-SLBP are caused, which is consistent with their smaller E_{ad}. Differently from NO_2 adsorption, SO_2 and CO_2 have no continuous orbital overlap in the single-electron defects of SV, DV3, EGz1 and EGa2. In all d-SLBP, they exhibited weaker orbital hybridization, which is supported by the smaller charge transfer (–0.11 to 0.01 e or –0.02 to 0 e).

In comparison with SO_2 or CO_2, the orbital overlap of NO and unsaturated d-SLBP is strong (Figure S8). Spin asymmetry was observed in the adsorption complex of NO formed with DV3, but not in the adsorption complexes formed with SV, EGz1 or EGa2. The adsorption complex of NO with EGz1 showed clear spin symmetry similar to that with SV or EGa2. However, the orbital overlap of NO with EGz1 was not as strong as that with SV or EGa2. The weak orbital hybridization was in accordance with the adsorption distance (1.98 Å, Figure S7c) between EGz1 and NO, which is slightly larger than the length of a P–N single bond. Additionally, the E_{ad} of EGz1 and NO are less negative than those of other unsaturated defects and NO. As for DV3 containing an even number of single electrons, the orbital symmetry of DV3 disappeared after being combined with NO. The adsorption complex of DV3 and NO showed obvious spin asymmetry near the Fermi level. According to PDOS, this spin asymmetry mainly comes from the defective phosphorus atoms of DV3.

S10. Experimental Details

Materials. Bulk black phosphorus (purity >99.998%) were bought from Nanjing XF NANO Materials Tech Co., Ltd. NO_2 gas was purchased from Beijing Lvyuan Dade Biological Tech Co., Ltd. (The certification of standard materials indicates that NO_2 was 200 ppm, and the rest was N_2.) High purity CO_2 and N_2 were obtained from Shenyang Shuntai Special Gas Co., Ltd. (The purity was greater than 99.5%.) Ultrapure water was obtained with a Millipore- Milli Q system.

Preparation Methods of LBP. LBP suspension was prepared by sonication of bulk black phosphorus in anaerobic water. Bulk black phosphorus was ground into a powder with an agate mortar in the glove box. The powder was dispersed in anaerobic water and sonicated for 12 hours. An ice bath was used to keep the temperature below 10 °C during sonication. Anaerobic water was prepared in a 250 mL anaerobic bottle continuously filled with N_2 while heating. The suspension was centrifuged at 10,000 rpm for 30 min.

Characterization and Analysis Methods. Raman spectroscopy (Thermo scientific DXR) was used to characterize LBP (Figure S27a). Three prominent peaks, i.e., A^1_g (out-of-plane phonon modes, assigned to 364 cm^{-1}), B_{2g} and A^2_g (in-plane modes, assigned to 439 and 466 cm^{-1}) are consistent with a previous report [17], confirming the successful exfoliation of LBP. A transmission electron microscope (TEM, JEM-2100F) was used to characterize the morphology of LBP. LBP has a thin layered structure with an obvious

edge structure. X-ray photoelectron spectroscopy (XPS, ESCALAB250, Thermo VG) was used to identify phosphorus components of LBP after treatment with N_2, NO_2 or CO_2.

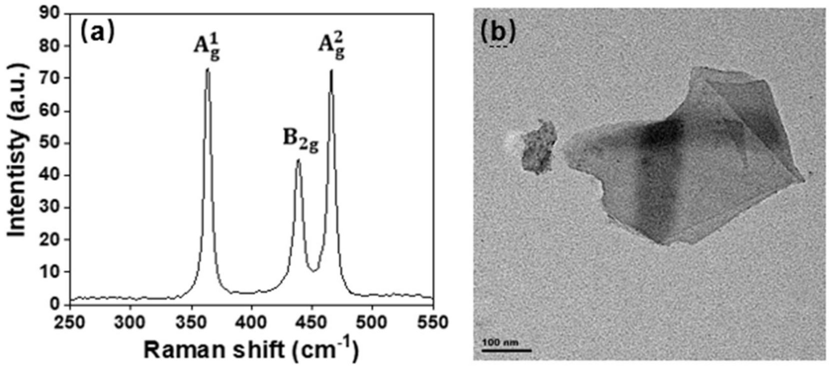

Figure S27. Characterizations of LBP. (a) Raman spectrum of LBP; (b) TEM image of LBP.

References

[1] Hiroshi, K.; Keiji, M.; Kenichi, F.; Teijiro, Y. The electronic structures of NO_2, NO_2^+ and NO_2^-. *Bull. Chem Soc Jpn* **1964**, 37, 11.

[2] Fourre, I.; Silvi, B. What can we learn from two-center three-electron bonding with the topological analysis of ELF? *Heteroatom Chem* **2007**, 18, 135-160.

[3] Frenking, G.; Loschen, C.; Krapp, A.; Fau, S.; Strauss, S.H. Electronic structure of CO--an exercise in modern chemical bonding theory. *J Comput Chem* **2007**, 28, 117-26.

[4] Cho, S.Y.; Lee, Y.; Koh, H.J.; Jung, H.; Kim, J.S.; Yoo, H.W.; Kim, J.; Jung, H.T. Superior chemical sensing performance of black phosphorus: Comparison with MoS_2 and graphene. *Adv. Mater.* **2016**, 28, 7020–7028.

[5] Zhang, Y.H.; Chen, Y.B.; Zhou, K.G.; Liu, C.H.; Zeng, J.; Zhang, H.L.; Peng, Y. Improving gas sensing properties of graphene by introducing dopants and defects: A first-principles study. *Nanotechnology* **2009**, 20, 185504.

[6] Kou, L.; Frauenheim, T.; Chen, C. Phosphorene as A superior gas sensor: Selective adsorption and distinct I-V response. *J. Phys. Chem. Lett.* **2014**, 5, 2675–2681.

[7] Cai, Y.; Ke, Q.; Zhang, G.; Zhang, Y.W. Energetics, charge transfer, and magnetism of small molecules physisorbed on phosphorene. *J. Phys. Chem. C* **2015**, 119, 3102–3110.

[8] Giannozzi, P.; Car, R.; Scoles, G. Oxygen adsorption on graphite and nanotubes. *J Chem Phys* **2003**, 118, 1003-1006.

[9] Yang, Q.; Chen, X.P.; Meng, R.S.; Jiang, J.-K.; Liang, Q.H.; Tan, C.J.; Cai, M.; Sun, X.; Yang, D.G.; Ren, T.L. First-principles study of sulfur dioxide sensor based on phosphorenes. *IEEE Electron Device Lett.* **2016**, 37, 660–662.

[10] Guo, S.; Yuan, L.; Liu, X.; Zhou, W.; Song, X.; Zhang, S. First-principles study of SO_2 sensors based on phosphorene and its isoelectronic counterparts: GeS, GeSe, SnS, SnSe. *Chem. Phys. Lett.* **2017**, 686, 83–87.

[11] Kaewmaraya, T.; Ngamwongwan, L.; Moontragoon, P.; Karton, A.; Hussain, T. Drastic improvement in gas-sensing characteristics of phosphorene nanosheets under vacancy defects and elemental functionalization. *J. Phys. Chem. C* **2018**, 122, 20186–20193.

[12] Wei, H.; Yang, J.Y. Defects in phosphorene. *J. Phys. Chem. C* **2015**, 119, 20474–20480.

[13] Farooq, M.U.; Hashmi, A.; Hong, J. Anisotropic bias dependent transport property of defective phosphorene layer. *Sci. Rep.* **2015**, 5, 12482.

[14] Banhart, F.; Arkady, V.K.; Kotakoski, J. Structural defects in graphene. *ACS Nano* **2011**, 5, 26–41.

[15] Liu, H.; Zhu, Z.; Luo, Z.; Xu, X.F.; Tomanek, D.; Ye, P.D. Phosphorene: An unexplored 2D semiconductor with A high hole mobility. *ACS Nano* **2014**, 8, 4033–4041.

[16] Mahabal, M.S.; Deshpande, M.D.; Hussain, T.; Ahuja, R. Sensing characteristics of phosphorene monolayers toward PH3 and AsH3 gases upon the introduction of vacancy defects. *J. Phys. Chem. C* **2016**, 120, 20428–20436.

[17] Zhao, Q.; Ma, W.; Pan, B.; Zhang, Q.; Zhang, X.; Zhang, S.; Xing, B. Wrinkle-induced high sorption makes few-layered black phosphorus: A superior adsorbent for ionic organic compounds. *Environ. Sci. Nano* **2018**, 5, 1454–1465.
